# Bayesian variable selection for parametric survival model with applications to cancer omics data

**DOI:** 10.1186/s40246-018-0179-x

**Published:** 2018-11-06

**Authors:** Weiwei Duan, Ruyang Zhang, Yang Zhao, Sipeng Shen, Yongyue Wei, Feng Chen, David C. Christiani

**Affiliations:** 1 Department of Biostatistics, School of Public Health, Nanjing Medical University, 101 Longmian Avenue, Nanjing, 211166 Jiangsu China; 2 China International Cooperation Center for Environment and Human Health, Nanjing Medical University, 101 Longmian Avenue, Nanjing, 211166 Jiangsu China; 3 Joint Laboratory of Health and Environmental Risk Assessment (HERA), Nanjing Medical University School of Public Health / Harvard School of Public Health, 101 Longmian Avenue, Nanjing, 211166 Jiangsu China; 4 Key Laboratory of Biomedical Big Data of Nanjing Medical University, 101 Longmian Avenue, Nanjing, 211166 Jiangsu China; 5 Department of Environmental Health, Harvard School of Public Health, Boston, MA USA; 6 Pulmonary and Critical Care Division, Department of Medicine, Massachusetts General Hospital/Harvard Medical School, Boston, MA 02114 USA

**Keywords:** Survival analysis, Bayesian variable selection, EM algorithm, Omics, Non-small cell lung cancer, Stomach adenocarcinoma

## Abstract

**Background:**

Modeling thousands of markers simultaneously has been of great interest in testing association between genetic biomarkers and disease or disease-related quantitative traits. Recently, an expectation-maximization (EM) approach to Bayesian variable selection (EMVS) facilitating the Bayesian computation was developed for continuous or binary outcome using a fast EM algorithm. However, it is not suitable to the analyses of time-to-event outcome in many public databases such as The Cancer Genome Atlas (TCGA).

**Results:**

We extended the EMVS to high-dimensional parametric survival regression framework (SurvEMVS). A variant of cyclic coordinate descent (CCD) algorithm was used for efficient iteration in M-step, and the extended Bayesian information criteria (EBIC) was employed to make choice on hyperparameter tuning. We evaluated the performance of SurvEMVS using numeric simulations and illustrated the effectiveness on two real datasets. The results of numerical simulations and two real data analyses show the well performance of SurvEMVS in aspects of accuracy and computation. Some potential markers associated with survival of lung or stomach cancer were identified.

**Conclusions:**

These results suggest that our model is effective and can cope with high-dimensional omics data.

**Electronic supplementary material:**

The online version of this article (10.1186/s40246-018-0179-x) contains supplementary material, which is available to authorized users.

## Introduction

With the development of high-throughput sequence technology, large-scale omics data are generated rapidly for discovering new biomarkers [1, 2]. The public databases such as The Cancer Genome Atlas (TCGA) and Gene Expression Omnibus (GEO) provide great opportunities to understand complex diseases comprehensively on a molecular level [3, 4] and subsequently facilitate growing demanding statistical approaches designed to cope with these large-scale data [5]. Analyzing biomarkers one at a time is the most common strategy to detect the underlying causal markers [6, 7]. However, this one-by-one method ignores the correlation between biomarkers and needs multiple corrections for controlling false positives. Furthermore, multiple regression is performed increasingly because it is powerful to identify causal markers after the strongest associations have been accounted for [8–10]. It can also avoid multiple correction and enable accurate effect estimation [11, 12]. Nevertheless, omics data generally have the property of high dimensionality, which makes the classical multiple regression yield unstable parameter estimations with high standard errors. Due to this limitation, least absolute shrinkage and selection operator (LASSO) regression and its variants shrink the effects of noise toward zero while adding a penalty term to the likelihood function. The approach can easily be conducted on a large-scale variable selection analysis [13, 14]. The LASSO can also be explained from Bayesian perspectives, namely Bayesian LASSO (BL) [15]. That is, we can generate the LASSO estimators by imposing a Laplace prior on coefficients of explanatory variables. Compared with a frequentist penalty, Bayesian regression is more flexible to induce different shrinkage by specifying various priors.

George and McCulloch [16] proposed a two-component “spike-and-slab” mixture prior, consisting of the “spike” to be either a point mass at zero or a normal distribution with a very narrow variance while a “slab” be a normal distribution with a large variance. Indicator variables are utilized to denote which component each marker belongs to, which is known as Bayesian variable selection (BVS). BVS and its expansions are widely used in genomic studies, including marker detection and disease risk prediction [11, 12, 17]. But, most Bayesian studies have used a Markov Chain Monte Carlo (MCMC) algorithm to explore the posterior distribution of unknown parameters via numerical approximations in high-dimensional models, which is quite time consuming for getting a stable chain. For example, the BayesR model required ~ 18 h to complete an analysis of a bipolar disorder genome-wide association data with ~ 3800 individuals (80% of whole samples) and ~ 300,000 genotype markers [12]. In order to facilitate Bayesian computation, variational inference [18–20] and expectation-maximization (EM) algorithm are commonly used for Bayesian posterior inference [21]. Ročková and George [22] proposed EM variable selection (EMVS) for continuous outcomes to rapidly identify promising high posterior models and parameter estimates. The continuous conjugate spike-and-slab prior adopted by EMVS leads to fast closed form expression for the EM algorithm. Nevertheless, there has been considerable interest in discovering associations between biomarkers and prognosis.

Analyzing time-to-event data, namely survival analysis, plays a very important role in statistics, which arises in many fields, such as medicine, genetics, industrial engineering, sociology, and economics [23–25]. Modeling survival data using Cox proportional hazards regression is popular for its robust to the unknown baseline hazard [26]. Alternatively, being well known for parametric survival analysis, accelerated failure time (AFT) model tends to give more precise estimates of interest parameters if the distribution of survival time is chosen correctly, in addition, the parameter estimates from AFT are robust to omitted covariates [27]. Under the scenario of high-dimensional survival analysis, a lot of works have been done usually by adding a penalty term to likelihood. In a Bayesian framework, we usually need to assign a semi-parametric or nonparametric prior processes to the (cumulative) baseline hazard function in a Cox model [28, 29], which does not allow us to naturally choose a fully parametric survival model for the subsequent analyses. As a parametric model, the Weibull regression induces a very flexible model since it is a unique parametric model which has both AFT and the proportional hazards properties [30].

In this study, we extended EMVS to parametric survival model (SurvEMVS) with Weibull distribution assumption. A fast EM algorithm was used to obtain posterior modes of interested parameters, in which a variant of the fast cyclic coordinate descent (CCD) method is nested. We used simulation trials to explore performance in comparison with an alternative frequentist variable selection strategy, namely Cox LASSO regression. After that, we applied SurvEMVS to a lung cancer genotype data and a stomach cancer gene expression data. Further details of this work were given below.

## Methods

### Statistical framework

Survival times of *n* individuals in sample are designed by *T*_*i*_ = min(*t*_*i*_, *c*_*i*_), *i* = 1, …, *n*, where *t*_*i*_ and *c*_*i*_ are lifetime and fixed censoring time for a specific individual *i*, respectively. The survival outcome from a follow-up study can be conveniently represented by pair of random variables (*T*_*i*_, *δ*_*i*_), where *δ*_*i*_ indicates whether the lifetime *t*_*i*_ corresponds to an event (*δ*_*i*_ = 1) or is censored (*δ*_*i*_ = 0). In this study, we consider right censoring scheme and non-informative censoring mechanism for each individual without note elsewhere. Under parametric framework, *f*(*T*_*i*_| *θ*) and *S*(*T*_*i*_| *θ*) are defined as probability density and survival function of survival time *T*_*i*_, respectively, parametrized by *θ*. Let $L={\prod}_{i=1}^nf{\left({T}_i|\theta \right)}^{\delta_i}S{\left({T}_i|\theta \right)}^{\left(1-{\delta}_i\right)}$ be the likelihood of the parametric model with an i.i.d assumption. Weibull distribution can be fully parametrized by the parameter pair *θ* = (*λ*, *α*), where *λ* and *α* are scale and shape parameter, respectively. The density and survival function of Weibull distribution *T* are *f*(*T*| *λ*, *α*) = *αT*^*α* − 1^*λ* exp(−*λT*^*α*^) and *S*(*T*| *λ*, *α*) = exp(−*λT*^*α*^), respectively. Typically in a regression model, the scale parameter is defined as *λ* = 1/ exp(*Zu* + *Xβ*), where *X*_*n* × *p*_ represents a column-scaled matrix of tumor biomarkers such as gene expression, genotype or DNA methylation, **β** is a *p* × 1vector of marker effects, *Z*_*n* × (1 + *q*)_is a covariates matrix of intercept and *q* clinical variables such as gender, age, and tumor histological grade, **u** is a (*q* + 1)-dimensional effect vector of *Z*_*n* × (1 + *q*)_. With the condition of *p* > *n*(i.e., high dimension), a penalize term is necessary for inducing a sparse solution of **β**. Under the Bayesian framework, we want to impose a well-known spike-and-slab prior on each *β*_*j*_ to facilitate Bayesian variable selection [16]. A vector of binary latent variables **γ** = (*γ*_1_, …, *γ*_*p*_)^*T*^, *γ*_*j*_ ∈ {0, 1} are introduced as indicator variables, where *γ*_*j*_ = 1 donates that *j*th explanatory variable is to be included in the model. Conditional on **γ**, the continuous prior being assigned to **β** is,

1 $$ \pi \left(\boldsymbol{\upbeta} |{\sigma}^2,\boldsymbol{\upgamma} \right)={\mathbf{N}}_p\left(\mathbf{0},{\mathbf{D}}_{\sigma^2,\boldsymbol{\upgamma}}\right), $$

where ${\mathbf{D}}_{\sigma^2,\boldsymbol{\upgamma}}={\sigma}^2\mathit{\operatorname{diag}}\left({a}_1,\dots, {a}_p\right)$and *a*_*j*_ = (1 − *γ*_*j*_)*υ*_0_ + *γ*_*j*_*υ*_1_. As suggested by [22], we set these hyperparameters *υ*_0_ and *υ*_1_ to be small and large positive values, respectively. *σ*^2^ is a common variance parameter with an inverse gamma prior *π*(*σ*^2^) = *IG*(*ν*/2, *νη*/2). The binomial prior is chosen for the indicator variable **γ**, i.e., $\pi \left(\boldsymbol{\upgamma} |\theta \right)=\prod \limits_{j=1}^p{\theta}^{\gamma_j}{\left(1-\theta \right)}^{1-{\gamma}_j}$, where *θ* is a hyperparameter and we impose a *Beta*(*a*, *b*) prior on it. The constant prior is chosen for *α* and **u**, i.e., *π*(*α*) ∝ 1 and *π*(**u**) ∝ 1.

Generally speaking, MCMC is usually used for simulating the posterior distribution of unknown parameters **β**, **γ**, *θ*, *σ*^2^, *α*, and **u**. But here we employ an EM algorithm to seek the posterior mode of each parameter because this algorithm provides substantial computational advantage, especially for high-dimensional data analysis. Concerning about the unknown status **γ** for variables, we replace this “missing data” by its conditional expectation given the current estimates for other parameters and observed data (E-step) [31]. Then, an M-step is followed by maximizing the expected complete-data log-posterior with respect to **β**, *θ*, *σ*^2^, *α*, and **u**. As a result of iterations between the E-step and M-step, each estimator will converge toward a local maximum of the posterior distribution. More specifically, the objective function can be expressed as:

2 $$ {\displaystyle \begin{array}{l}Q\left(\upbeta, \theta, {\sigma}^2,\alpha, \mathbf{u}|T,\delta \right)={E}_{\upgamma \mid \cdot}\left[\log\ \pi \left(\upbeta, \upgamma, \theta, {\sigma}^2,\alpha, \mathbf{u}|T,\delta \right)\right]\\ {}\kern10.8em =C+\log\ L+{E}_{\upgamma \mid \cdot}\left[\log\ \pi \left(\upbeta |{\sigma}^2,\upgamma \right)\right]+{E}_{\upgamma \mid \cdot}\left[\log\ \pi \left(\upgamma |\theta \right)\right],\\ {}+\log\ \pi \left(\theta \right)+\log\ \pi \left({\sigma}^2\right)+\log\ \pi \left(\alpha \right)+\log\ \pi \left(\mathbf{u}\right)\end{array}} $$

where *E*_**γ** ∣ ⋅_(⋅) denotes the expectation with regard to **γ** given estimations in current iteration. Furthermore, ${E}_{\boldsymbol{\upgamma} \mid \cdot}\left[\log \pi \left(\boldsymbol{\upbeta} |{\sigma}^2,\boldsymbol{\upgamma} \right)\right]={C}_1-\frac{p}{2}\log {\sigma}^2-\frac{1}{2{\sigma}^2}{\sum}_{j=1}^p{\beta}_j^2{E}_{\boldsymbol{\upgamma} \mid \cdot }{\left[\left(1-{\gamma}_j\right){\upsilon}_0+{\gamma}_j{\upsilon}_1\right]}^{-1}$, and ${E}_{\boldsymbol{\upgamma} \mid \cdot}\left[\log \pi \left(\boldsymbol{\upgamma} |\theta \right)\right]={\sum}_{j=1}^p{E}_{\boldsymbol{\upgamma} \mid \cdot}\left[{\gamma}_j\right]\log \left(\frac{\theta }{1-\theta}\right)+p\log \left(1-\theta \right)$, both *C* and *C*_1_ are constants. Next, our EM algorithm for Bayesian Weibull regression proceeds as follows.

1. Initialize the unknown parameters: **β**^(0)^, *θ*^(0)^, *σ*^2(0)^, *α*^(0)^, **u**^(0)^.
2. E-step:

As can be seen from the formula (2) above, there are two parts that need further evaluation, namely *E*_**γ** ∣ ⋅_[*γ*_*j*_] and *E*_**γ** ∣ ⋅_[(1 − *γ*_*j*_)*υ*_0_ + *γ*_*j*_*υ*_1_]^−1^. In particular, *E*_**γ** ∣ ⋅_[*γ*_*j*_] is a conditional expectation of *γ*_*j*_ and depends on observed data (*T*, *δ*) only by means of current parameter estimates **β**^(*k*)^, *θ*^(*k*)^, *σ*^2(*k*)^ because of the hierarchical structure of **γ**; therefore, we have

3 $$ {E}_{\boldsymbol{\upgamma} \mid \cdot}\left[{\gamma}_j\right]=P\left({\gamma}_j=1|{\boldsymbol{\upbeta}}^{(k)},{\theta}^{(k)},{\sigma}^{2(k)}\right)={p}_j^{\ast }, $$

where $$ {p}_j^{\ast }=\frac{\pi \left({\beta}_j^{(k)}|{\sigma}^{2(k)},{\gamma}_j=1\right)P\left({\gamma}_j=1|{\theta}^{(k)}\right)}{\pi \left({\beta}_j^{(k)}|{\sigma}^{2(k)},{\gamma}_j=1\right)P\left({\gamma}_j=1|{\theta}^{(k)}\right)+\pi \left({\beta}_j^{(k)}|{\sigma}^{2(k)},{\gamma}_j=0\right)P\left({\gamma}_j=0|{\theta}^{(k)}\right)}, $$

meanwhile, the second part will be easily derived as

4 $$ {E}_{\boldsymbol{\upgamma} \mid \cdot }{\left[\left(1-{\gamma}_j\right){\upsilon}_0+{\gamma}_j{\upsilon}_1\right]}^{-1}=\frac{1-{p}_j^{\ast }}{\upsilon_0}+\frac{p_j^{\ast }}{\upsilon_1}={d}_j^{\ast }. $$

(3) M-step:

Next, we derive the M-step for the objective function *Q*(**β**, *θ*, *σ*^2^, *α*, **u**| *T*, *δ*).

1. Differentiating *Q*(⋅| *T*, *δ*) with regard to **β** needs to solve the following expression

5 $$ {\boldsymbol{\upbeta}}^{\left(k+1\right)}=\arg {\min}_{\boldsymbol{\upbeta}}\left\{-\log L+\frac{1}{2{\sigma}^2}{\left\Vert {\mathbf{D}}^{\ast 1/2}\boldsymbol{\upbeta} \right\Vert}^2\right\}, $$

where **D**^∗1/2^ is the square root of the *p* × *p* diagonal matrix ${\mathbf{D}}^{\ast }=\mathit{\operatorname{diag}}\left({d}_1^{\ast },\dots, {d}_p^{\ast}\right)$. It can be shown that the formula (5) above for Weibull model is convex, and a wide variety of numerical optimization algorithms can be applicable. For generalizing our algorithm to high-dimensional data, the commonly used multidimensional Newton-Raphson method is not recommended because of large memory requirements and intensive computations. In this research, we employ the cyclic coordinate descent (CCD) algorithm due to its efficiency and ease of implementation, which makes one-dimensional optimization available [32]. Briefly, the CCD minimizes the objective function with regard to *β*_*j*_, holding all other variables constant. Similar to the combined local and global (CLG) algorithm of [33, 34], we modify the update for *β*_*j*_ in two ways. First, in order to avoid big steps in Newton iteration, we specify a positive value Δ_*j*_ for the *j*th maker to restrict the maximum change of *β*_*j*_ between two adjacent iterations. Second, for one-dimensional optimization, we update *β*_*j*_ only once instead of multiple iterations till convergence before updating *β*_*j* + 1_. Moreover, considering that it is unnecessary to take much time to update the amount of possible neural markers (that is, their minuscule effects contribute less to outcome, with no need for accurate estimations) when *p* is large, we partially update those markers with “large” effect (|*β*_*j*_|is greater than a threshold *β*^′^, e.g., 1E−08) after a small number (*k*^′^) of full iterations for all makers, which speeds up computation considerably.

2) Differentiating *Q*(⋅| *T*, *δ*) with regard to *u*_*j*_, *j* = 1, …, *q* + 1one-by-one using one-dimensional optimization reveals the following form

6 $$ {u}_j^{\left(k+1\right)}\left|{}_{{\boldsymbol{\upbeta}}^{\left(k+1\right)},\alpha, {\sigma}^2,\theta, {u}_{j^{-}}^{\left(k+1\right)},{u}_{j^{+}}}\right.={u}_j-\frac{\partial \log L}{\partial {u}_j}{\left(\frac{\partial^2\log L}{\partial {u}_j^2}\right)}^{-1}, $$

with all other parameters being fixed at current estimates.

3) Differentiating *Q*(⋅| *T*, *δ*) with regard to *α* derives the following form

7 $$ {\alpha}^{\left(k+1\right)}\left|{}_{{\boldsymbol{\upbeta}}^{\left(k+1\right)},{\mathbf{u}}^{\left(k+1\right)},{\sigma}^2,\theta}\right.=\alpha -\frac{\partial \log L}{\partial \alpha }{\left(\frac{\partial^2\log L}{\partial {\alpha}^2}\right)}^{-1}. $$

4) For *σ*^2^, we have

8 $$ {\sigma}^{2\left(k+1\right)}=\frac{\sum_{j=1}^p{\beta}_j^{\left(k+1\right)2}{d}_j^{\ast }+\nu \eta}{p+\nu +2}. $$

5) For *θ*, we have

9 $$ {\theta}^{\left(k+1\right)}=\frac{\sum_j^p{p}_j^{\ast }+a-1}{a+b+p-2}. $$

(4) Iterations between the E-step and M-step are in progress. SurvEMVS will be terminated if the convergence criterion is satisfied as follows: ${\sum}_{i=1}^n\left|{\mathbf{X}}_i^T\left({\boldsymbol{\upbeta}}^{(k)}-{\boldsymbol{\upbeta}}^{\left(k-1\right)}\right)\right|/\left(1+{\sum}_{i=1}^n\left|{\mathbf{X}}_i^T{\boldsymbol{\upbeta}}^{(k)}\right|\right)<\xi$, where **Xβ**^(*k*)^ is predictive vector at *k*th iteration, and *ξ* is a small value (say 10^− 4^). Summarizing, Additional file 1: Figure S1 presents pseudocode for our implementation of SurvEMVS.

### Simulation studies

In this section, we used simulations to validate the performance of proposed SurvEMVS. Cox LASSO model [35] was considered as a benchmark for comparison. The effect sizes and directions of Cox LASSO estimates were adjusted for consistency with our parametric model, which made the direct comparison between two methods. For each simulation scenario, we replicated the simulation 50 times and then summarized these results.

Marker values were simulated from a multivariate normal distribution N_50_(0, ∑), where ∑ is a variance-covariance symmetric matrix with ∑_*jj*_ = 1 and ∑_*ij*_ = 0.6^|*i* − *j*|^, *i* ≠ *j*. For large *p* markers, we repeatedly sampled from the above distribution and then combined them by column. Thus, we obtained an *n* × *p* matrix with multiple independent blocks and 50 makers in each block were correlated. Assuming ${\lambda}_i=\exp \left(-{\sum}_j^p{x}_{ij}{\beta}_j\right)$, we simulated survival time *t*_*i*_ for each subject from an exponential distribution *t*_*i*_~exponential(*λ*_*i*_) , and random censoring time *c*_*i*_ from a uniform distribution *c*_*i*_~*U*(0, K), and chose K such that on average 40% subjects were right censored. The observed censored survival time *T*_*i*_ was generated by min(*t*_*i*_, *c*_*i*_). Furthermore, additional simulations where survival times are generated from Weibull distribution (with *α* = 2) were used to show effectiveness of our method. As is well-known, the true distribution of survival time in a real data is unclear and does not coincide with the Weibull assumption exactly. Therefore, we simulated a vector of Gamma-distributed survival time on purpose, thus assigning weakness setting to SurvEMVS. The shape and rate parameters of gamma distribution were set to be 0.8 and 1/*λ*_*i*_, respectively. We randomly sampled six causal markers and set coefficients to be {0.2,   − 0.2,  0.3,   − 0.3,  0.4,   − 0.4}. Sample size (*n*) was set to be 500, and number of makers is set to be 1000 or 5000. Moreover, we generated 100 samples as a test dataset in each replication to appreciate the predictive performance of two approaches. The detailed simulation scenarios were summarized in Table 1.

**Table 1:** Parameters settings for simulation studies

Parameter	Scenario					
	1	2	3	4	5	6
Censoring rate	40%					
Causal effects	− 0.2, 0.2, − 0.3, 0.3, − 0.4, 0.4					
Replications	50					
Sample size of test dataset	100					
Distribution of survival time (shape)	Exponential		Weibull (2)		Gamma (0.8)	
Sample size (*n*) / No. of makers(*p*)	500/1000	500/5000	500/1000	500/5000	500/1000	500/5000

### Hyperparameters tuning and performance metric

The performance of SurvEMVS depended on the hyperparameters *υ*_0_ and *υ*_1_, which made us be interested in a running model based on more than one combination of *υ*_0_ and *υ*_1_. The speed of the EM algorithm made it feasible to consider a sequence of models as (*υ*_0_, *υ*_1_) varied over many candidates, from which we could select an optimal combination based on some criteria. According to [22], large *υ*_1_ and small *υ*_0_ could accommodate all plausible *β*_*j*_. In order to acquire sparse solution in large *p* data, we set a sequence of candidates {1/10*p*, 1/5*p*, 1/2*p*, 1/*p*, 2/*p*, 5/*p*, 10/*p*, 0.05} (0.05 was reserved if *p* > 500) to *υ*_0_ unless otherwise noted, which was dynamic with *p*. Three candidates from {10,  100,  500} were assigned to *υ*_1_. Therefore, there were 24 combinations for hyperparameter tuning. The procedure of fitting Cox LASSO by widely used R package *glmnet* gave the similar parameter tuning as we did here.

On account of making parameter selection from the 24 combinations of *υ*_0_ and *υ*_1_, we needed a metric to measure the performance of the fitted models. Cross-validation partial likelihood (CVPL) was generally used for parameter selection in Cox model [35, 36], while some employed cross-validation score in parametric survival model [36]. Subsequently, one candidate of hyperparameters was chosen to minimize one of these metrics. However, these metrics involving random cross-validation (CV) make the hyperparameter selection not stable as well as time demanding, and depend on the folds. Other criteria without CV like the Akaike information criterion (AIC), the Bayesian information criterion (BIC), and the generalized cross-validation (GCV) tend to select many spurious variables especially in high-dimensional problem [37, 38]. In this paper, we considered a metric, namely extended Bayesian Information Criteria (EBIC), which was utilized for model selection at first [39]. The constant prior behind the BIC assigns high probabilities to the models with a larger number of markers, which was apparently unreasonable and strongly against the principle of parsimony. The EBIC was proposed to take away this disadvantage of the BIC. The EBIC is defined as,

10 $$ {\mathrm{EBIC}}_{\gamma }=-2\log L+{p}_m\log n+2\tau \left(\begin{array}{c}p\\ {}{p}_m\end{array}\right),0\le \tau \le 1, $$

where *p*_m_ is the number of selected variables in a fitted model. The EBIC with *τ* = 0 reduces to the original BIC. Minimizing the EBIC with larger *τ* will get a much more parsimonious model. Thus, EBIC1, EBIC2, and EBIC3 were served as metrics for hyperparameter tuning with regard to *τ* = 0,  0.5,  1, respectively.

In simulation studies, the optimal SurvEMVS model selected by the EBIC was compared with Cox LASSO in aspects with variable selection, effect estimation, and model prediction. We utilized false positive rate (FPR), true positive rate (TPR), and false discovery rate (FDR) as evaluation indicators for variable selection. Note that $P\left({\gamma}_j=1\left|{\widehat{\beta}}_j,\widehat{\theta},{\widehat{\sigma}}^2\right.\right)\ge 0.5$ meant the *j*th maker was selected. Mean square error (MSE) denoted by ${\sum}_{j=1}^p{\left({\widehat{\beta}}_j-{\beta}_j\right)}^2/p$ was used to appreciate effect estimations for makers. The predictive accuracy of the fitted model be applied to test dataset was evaluated by Harrell’s *c* statistic [40], as known as the area under the ROC curve (AUC).

### Implementation

We considered *a* = *b* = 1 in beta prior for *θ* which yielded a uniform distribution. As noted in [22], we had inverse gamma prior for *σ*^2^ with *ν* =* η* = 1 to make this prior relatively non-influential. We ran all analyses using R software (v3.41) on a machine with Intel® Xeon® X5690 3.46-GHz processors. Cox LASSO model was implemented with the *glmnet* package in R. Tenfold cross-validation was used to choose an optimal penalization parameter *λ*_*lasso*_ in *glmnet*, which determined an optimal Cox LASSO model. Two LASSO models selected by “minimum cvm” and “1 standard error” of *λ*_*lasso*_ were considered as LASSO.min and LASSO.se, respectively. We employed the PLINK tool for quality control of genotype data [41].

## Results

### Simulation studies

#### Iteration and tuning plot

**Fig. 1:**
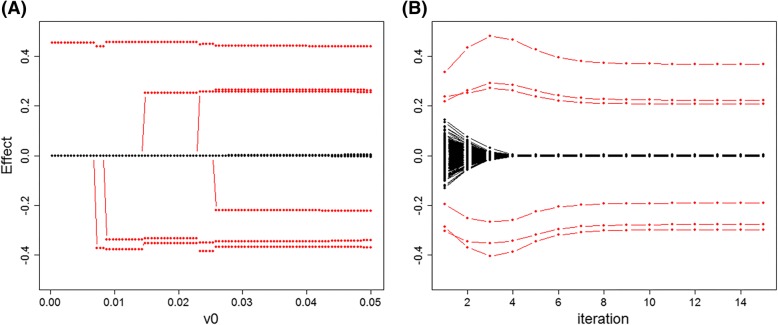
Solution path and iteration path of the proposed SurvEMVS under Scenario 1. Red dots represent the changes of estimated effects for the true signals. **a** Solution path. **b** Iteration path (*υ*_0_ = 0.05)

By analogy with LASSO solution path plot that shows the estimates change with an increasing penalty parameter, here we want to investigate the impact of parameters tuning for *υ*_0_. Figure 1a displays a solution path of SurvEMVS under Scenario 1 with *υ*_1_=10. Large effects (red dots) will be firstly incorporated into the fitting model with *υ*_0_ increasing, and a remarkable separation between the positive and negative effects appears when *υ*_0_ is larger than 0.03. However, the estimated effects for zeros inflate because of less shrinkage at large *υ*_0_. We also present the iteration plot to detect the convergence property of our approach. From Fig. 1b, SurvEMVS makes a fast convergence to posterior estimates only in several steps. Moreover, neutral effects (black dot line) get close to zero in the fourth iteration, which means that we can concentrate iterations on large effects after a number of full iterations for all makers and further accelerate the posterior computation.

#### Variable selection

In SurvEMVS, conditional posterior probabilities are used to guide variable selection. Table 2 shows comparison results between SurvEMVS and Cox LASSO in variable selection for Scenarios 1 and 2. For Scenario 1 with *p* = 1,000, all models except LASSO.min can reduce noise markers with low FDRs, and all of three models with regard to SurvEMVS acquire high TPRs. When simulated *p* increases to 5000 (Scenario 2), FPR and FDR of BIC (i.e., EBIC1) inflate seriously. These indicate that proper extra penalty on the BIC of SurvEMVS brings to a moderate result of variable selection. We summarize the results of Scenarios 3 and 4 with Weibull distribution in Additional file 1: Table S1, and the results of Scenarios 5 and 6 with gamma distribution in Additional file 1: Table S2. Each of them presents a similar trend with scenarios of exponential distribution.

**Table 2:** TPR, FPR, and FDR in variable selection with 50 replications (exponential distribution)

Method	Scenario 1 (*p* = 1000)			Scenario 2 (*p* = 5000)		
	TPR	FPR	FDR	TPR	FPR	FDR
LASSO.se	0.657	1.25E−03	0.239	0.327	1.36E−04	0.258
LASSO.min	0.920	2.11E−02	0.792	0.713	4.60E−03	0.843
EBIC (*τ* = 0)	0.743	1.19E−03	0.209	0.703	5.78E−03	0.872
EBIC (*τ* = 0.5)	0.730	7.85E−04	0.151	0.480	1.48E−04	0.204
EBIC (*τ* = 1.0)	0.710	6.24E−04	0.127	0.377	2.00E−05	0.042

Abbreviations: TPR, true positive rate; FPR, false positive rate; FDR, false discovery rate

#### Parametric estimation

**Fig. 2:**
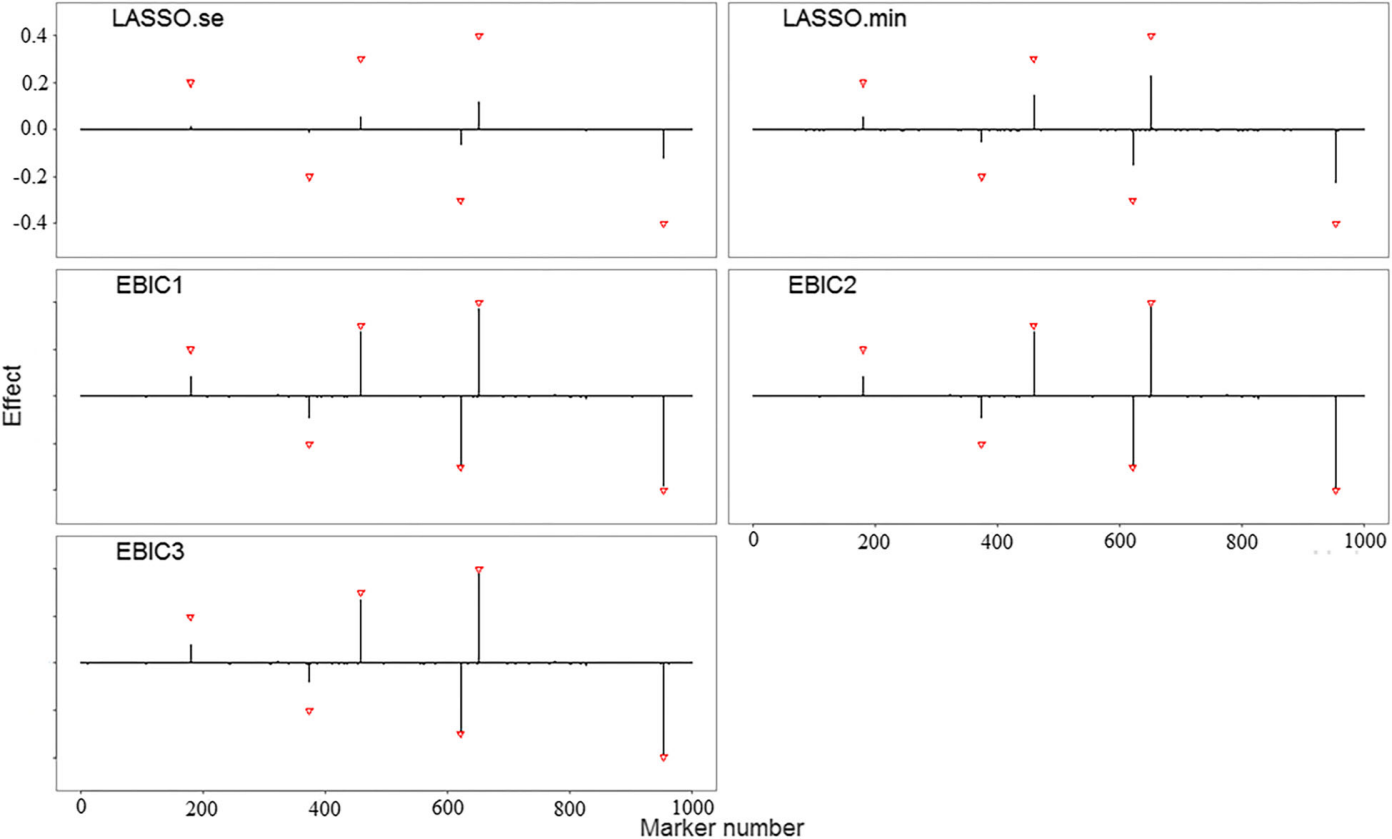
Averaged estimated effect (*black vertical lines*) for each marker over 50 replications under Scenario 1. Red triangles label true effect sizes and locations of the causal markers

**Fig. 3:**
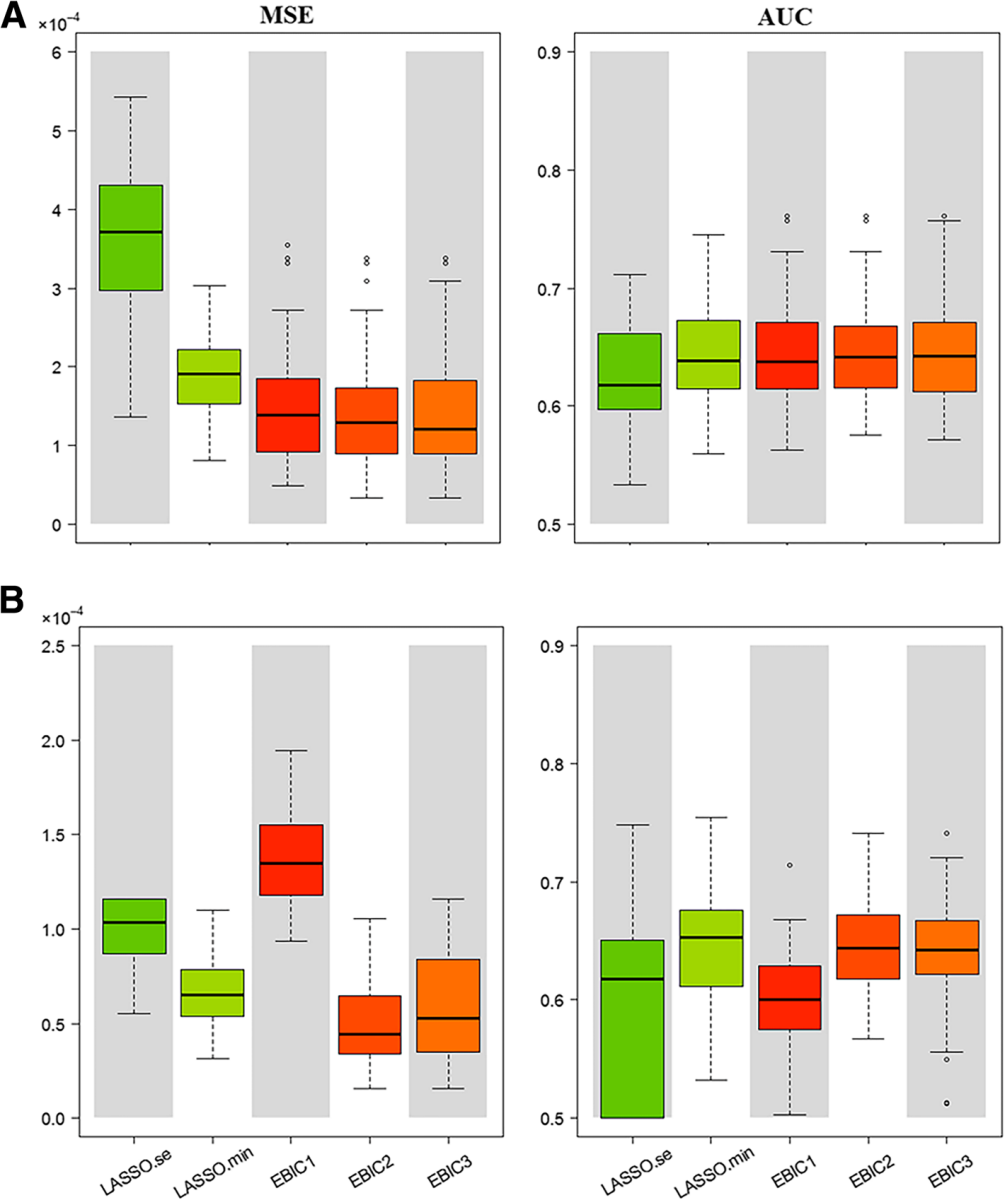
MSE of parameter estimation and AUC of prognosis prediction for Scenarios 1 and 2. **a**, **b** The results of Scenarios 1 and 2, respectively

Figure 2 and Additional file 1: Figures S2-S6 show the parameter estimations of five models for all scenarios. Averaged estimated effects (*black vertical lines*) of estimated effects for all makers are calculated over 50 trials. Triangles in all figures label the locations and effect sizes of the pre-specified causal makers. In all scenarios, SurvEMVS gives a lower bias than Cox LASSO. Biases of all models increase with number of variants. Two models of SurvEMVS (i.e., EBIC2 and EBIC3) present a similar estimator, while the estimated effects in EBIC1 for zeros inflate under scenarios with *p* = 5,000 (rough *X*-axis in Additional file 1: Figures S2, S4 and S6). In order to make a comprehensive evaluation of bias and variance, we use the MSE metric and present the results in the left panels of Fig. 3 and Additional file 1: Figures S7 and S8. Both the EBIC2 and EBIC3 are well performed under all scenarios, whereas the EBIC1 model get a high MSE with *p* = 5,000. There is no apparent difference between the results of exponential, Weibull, and gamma distribution.

#### Prediction accuracy

In order to appreciate prediction accuracy of the fitted models, we summarize AUC results by box plot in the right panels of Fig. 3 and Additional file 1: Figures S7 and S8. Generally speaking, the EBIC2 model performs best under our simulation settings, while the LASSO.min presents similar prediction but with larger variance. In accordance with the conclusion of “Parametric estimation”, prediction accuracy of the BIC model descends with *p* varying from 1000 to 5000. Moreover, SurvEMVS with exponential or Weibull settings gain slightly larger AUC than those with the gamma settings. Furthermore, the LASSO.se model almost provides the lowest AUC among simulation scenarios. All the above results indicate that the BIC is not suitable for large *p* scenario.

In summary, the EBIC2 model works best under almost all scenarios in terms of variable selection, parameter estimation, and prediction accuracy. Besides, Additional file 1: Table S3 shows the time consumption in different scenarios. In comparison with Cox LASSO, SurvEMVS takes more computational time but is still fast enough. Note that time used in Additional file 1: Table S3 is for reference only, as it varies depending on context, such as convergence criterion, programming language, computer performance, and algorithm optimization.

### Real data analysis

#### Harvard lung cancer data

This dataset from The Harvard Lung Cancer Susceptibility Study GWAS includes 526 late-stage (III and IV) patients with non-small cell lung cancer (NSCLC) recruited from Massachusetts General Hospital (Boston, MA). More details about participants’ recruitment have been described previously [42]. We note that it is appropriate to assess an association study restricted to late-stage cancer because some gene functions work primarily in the late stage and are not present in preinvasive stages of cancer [43]. DNA was genotyped using Illumina 610K Quad chip. After quality control protocol described by [44], there were 512,885 SNPs remaining. Those patients with more than 5 years of overall survival were considered as right censored, and finally, the censor rate was equal to 20.27%. We assumed an additive genetic model and imputed missing genotypes by mean of each SNP. We adjusted for age, sex, smoking status, cell type, stage, surgery (yes vs. no), and the top four principal components in the subsequent analysis. Considering that the number of SNPs related to NSCLC survival was not expected to be too large, we filtered the SNPs by a commonly used single locus Cox model. This filter yielded a de-noising of outcome so that the subsequent analyses became more efficient. By setting a threshold of *P* value less than 5E−3, 3911 SNPs were left for the subsequent analysis.

The EBIC2 was used to choose an optimal model from the candidates we noted above. Finally, 14 SNPs were detected by the proposed EBIC2 model with *υ*_1_ = 100 and υ_0_=2.56E-03. We further analyzed and annotated these SNPs by TCGA (with an online tool UALCAN [45]), KEGG pathway, and PubMed database. Interestingly, seven of all listed in Table 3 may have potential functional influence on carcinogenesis or prognosis. For example, rs1506943 is located at ~ 33 kb downstream of RXRG (retinoid X receptor gamma) on Chromosome 1. This gene is expressed at significantly lower levels in TCGA-LUAD (lung adenocarcinoma) and TCGA-LUSC (lung squamous cell carcinoma) tumor samples and samples of other research [46]. Additionally, it also participates in non-small cell lung cancer pathways and other cancer-related pathways. rs2074986 is located at DNase I hypersensitive site (DHS) of GFRA1 on Chromosome 10. GFRA1 released by nerves enhances cancer cell perineural invasion [47], whose expression is reduced in tumor samples of TCGA-LUAD and TCGA-LUSC compared with normal samples. In addition, the high expression level in TCGA-LUAD tumor samples may contribute to good prognosis (*P* = 0.0025). We also provided the estimated effects of 14 SNPs by SurvEMVS in Additional file 1: Table S4 along with classical Weibull regression estimations for them (that is, only 14 SNPs and clinical variables are fitted by Weibull regression). In this way, SurvEMVS applied in high-dimensional data can also generate approximate estimates with Weibull regression. We plotted Kaplan-Meier (KM) survival curve of patients with high, moderate, and low risk defined by tertiles of risk scores $-{\sum}_j^p{x}_{ij}{\beta}_j$ (Additional file 1: Figure S9). The log-rank test was used to compare the survival estimates among the three groups, and the results show that higher prognostic risk score is significantly associated with shorter survival (*P* < 1E−16). However, Cox LASSO models did not identify any SNP.

**Table 3:** Validation analysis of the seven potential SNPs identified by SurvEMVS using external database

SNP (Cytoband)	Gene symbol (annotation)	TCGA	KEGG	PubMed
rs1506943_G (1q23.3)	LMX1A-RXRG (Intergenic)	RXRG: low expression in LUAD and LUSC tumor samples	RXRG participates in non-small cell lung cancer pathway and other cancer related pathways (hsa05200, 05222)	This gene is expressed at significantly lower levels in non-small cell lung cancer cells [46].
rs1921660_G (2q37.3)	GBX2-ASB18 (Intergenic)	GBX2: high expression is associated with bad prognosis (*P* = 0.0052)	–^a^	Enhanced GBX2 expression stimulates growth of human prostate cancer cells [64, 65].
rs981852_C (3p14.2)	FHIT (Intron)	Low expression in LUSC tumor samples	FHIT participates in non-small cell lung cancer pathway and Small cell lung cancer pathway(hsa05222, 05223)	–
rs2044831_G (7p14.1)	EPDR1 (Coding)	Low expression in LUSC tumor samples	–	EPDR1 is highly expressed in colorectal tumor cells [66].
rs263264_G, (8q24.2)	ADCY8 (Intron)	–	ADCY8 participates multiple signal pathways and pathways in cancer	–
rs2074986_G (10q25.3)	GFRA1 (DHS)	Low expression in LUAD and LUSC tumor samples; High expression in LUAD is associated with good prognosis (*P* = 0.0025)	–	GFRA1 released by nerves enhances cancer cell perineural invasion [47]; Methylation changes of GFRA1 may be a potential biomarker for prediction of gastric carcinoma metastasis [67].
rs4885110_A (13q22.1)	LINC00393-KLF12 (Intergenic)	KLF12: low expression in LUAD tumor samples	–	KLF12 is an important regulator of gene expression during carcinogenesis [68–70].

^a^Negative results of validation analysis

#### TCGA stomach adenocarcinoma (TCGA-STAD) expression data

We accessed this RNA-seq transcriptomic data from TCGA database by R/Bioconductor package *TCGAbiolinks* [48], which was used to do subsequent quality control, normalization, differential expression analysis (DEA), and visualization. The clinical information is summarized in Additional file 1: Table S5. Similar to the first real data analysis, we built final models only using filtered expression markers by DEA rather than all markers. There were 2711 markers left passing a selection threshold defined at fold change (FC) > 2 and testing FDR < 0.01. Due to the relative high missing rate, we made use of multivariate imputation by chained equations (MICE) to deal with missing clinical covariates [49]. After removing the patients with missing or zero survival time, we apply SurvEMVS and Cox LASSO to a matrix with 390 rows and 2716 columns (including 5 clinical covariates listed in Additional file 1: Table S5).

**Fig. 4:**
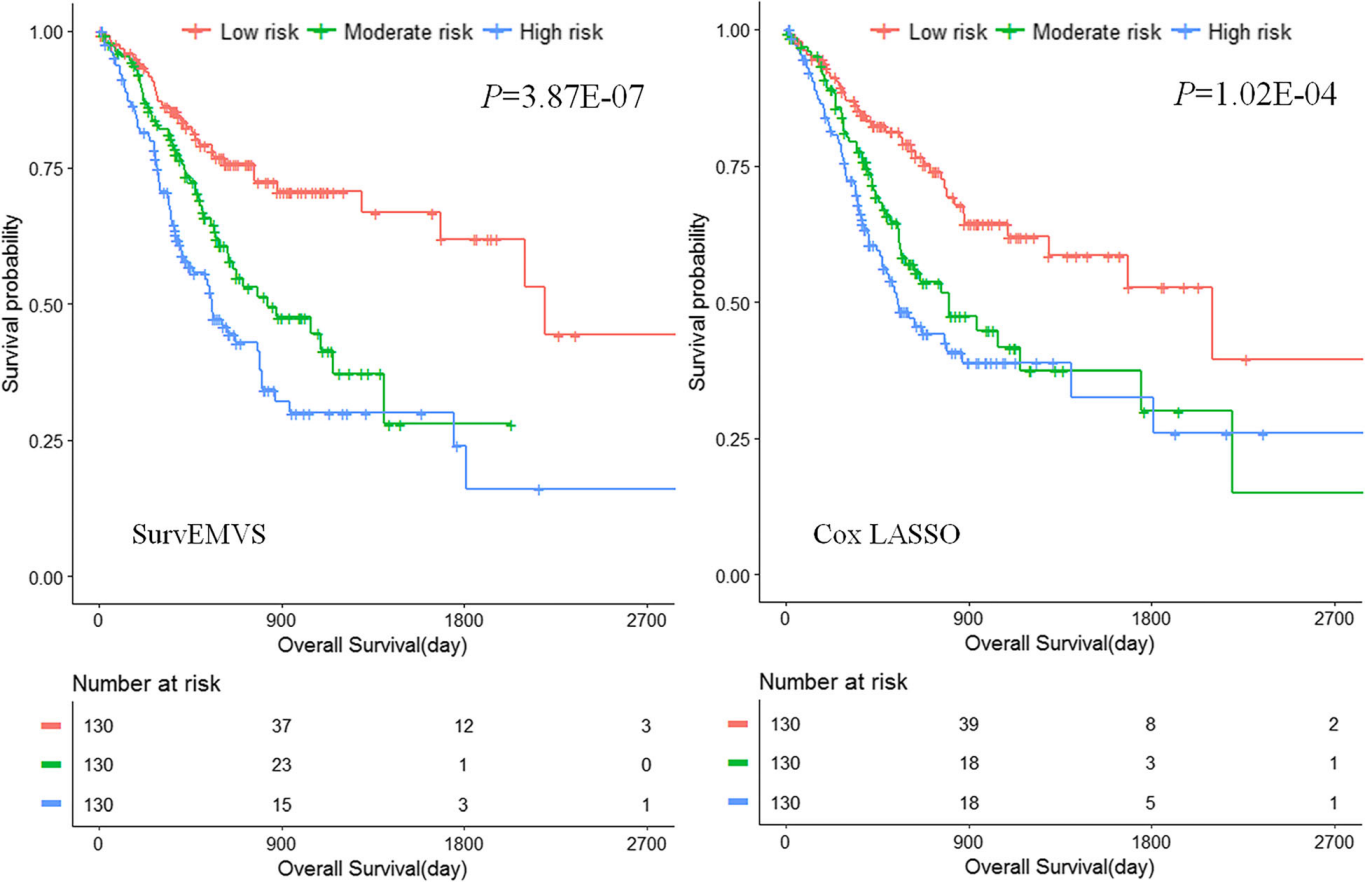
Kaplan-Meier survival curve of patients with high, moderate, and low risk. *P* value is calculated using log-rank test

We used the EBIC2 to select best model from the candidates above. Three markers were identified by the proposed model (*υ*_0_ = 3.69E-03 and *υ*_1_ = 500) including CTLA4, NACAD, and SERPINE1, mapped to 2q33.2, 7p13, and 7q22.1, respectively. Meanwhile, the LASSO.min detected ALG11, GAMT, and PLCXD3 in addition to the overlap genes CTLA4 and SERPINE1. Estimated effects of the selected markers in both two models are provided (Additional file 1: Table S6) along with classical Weibull regression and Cox model estimations for them (like the first application). We can see that many effects estimated by Cox LASSO are small while SurvEMVS presents similar estimations with its counterpart in the low-dimensional Weibull regression. According to tertiles of risk scores, we equally divided the patients into high-, moderate-, and low-risk groups. Figure 4 presents the KM curves of SurvEMVS (left panel) and Cox LASSO (right panel), respectively, and both of them show a higher risk score which is significantly associated with shorter survival (Log-rank test *P* = 3.87E−07 for SurvEMVS and *P* = 1.02E−04 for Cox LASSO).

Furthermore, in order to validate the results, we used external data from GEO database. Five datasets (GSE14210, GSE15459, GES29272, GSE51105, and GSE62254) are included for their proper sample sizes. Table 4 presents the estimated hazard ratios along with 95% confidence intervals (CI) and *P* value extracted from an online tool (KM plotter, *Web resource*). We also show the combined results using meta-analysis. As a result, CTLA4 and NACAD were successfully validated and have the same direction of the effects on prognosis with those estimated by SurvEMVS in TCGA-STAD data. Interestingly, CTLA4 encodes CTLA-4 (cytotoxic T-lymphocyte-associated protein 4) which inhibits T cell activation and downregulates immune response. Antagonistic antibody against CTLA has become a targeted drug (Ipilimumab, approved by FDA for melanoma in 2011), which is the first approved and popular immune checkpoint blockade therapy [50, 51]. Some research also indicates CTLA4 may have influence on gastric cancer germination and progression [52, 53]. GAMT and PLCXD3 detected by Cox LASSO display strong heterogeneity on effects (81.8% and 82.1%, respectively) with five datasets and no significant association in the combined analysis. Note that ALG11 is not identified in the five data. Although we acquire a negative consequence of SERPINE1 (also known as plasminogen activator inhibitor-1, PAI-1) in validation analysis; interestingly, it has been widely studied and is well known for participating p53 signaling pathway and playing a crucial role in tumor progression and angiogenesis [54, 55].

**Table 4:** Validation analysis of five genes using five GEO datasets

Gene symbol	GSE14210 (*N* = 119)	GSE15459 (*N* = 197)	GSE29272 (*N* = 126)	GSE51105 (*N* = 93)	GSE62254 (*N* = 283)	Heterogeneity-*P* (*I*^2^)	Combined (random)
CTLA4 (2q33.2)	–^a^	0.77 (0.52~1.12) 1.7E−01	–	0.45 (0.25~0.82) 7.6E−03	0.59 (0.41~0.85) 4.1E−03	3.0E−01 16.9%	0.62 (0.48~0.82) 5.6E−04
PLCXD3 (5p13.1)	–	0.77 (0.51~1.15) 2.0E−01	–	1.60 (0.98~2.6) 5.8E−02	1.92 (1.32~2.79) 5.0E−04	4.0E−03 82.1%	1.33 (0.75~2.36) 3.3E−01
NACAD (7p13)	1.47 (0.97~2.24) 7.1E−02	1.47 (0.93~2.34) 9.8E−02	1.61 (1.04~ 2.49) 3.0E−02	1.76 (1.06~2.92) 2.7E−02	2.03 (1.41~2.92) 9.3E−05	7.7E−01 0.0%	1.68 (1.39~2.04) 1.2E−07
SERPINE1 (7q22.1)	1.46 (0.99~2.16) 5.6E−02	0.83 (0.56~1.24) 3.7E−01	0.73 (0.48~ 1.10) 1.3E−01	1.27 (0.76~2.12) 3.7E−01	1.57 (1.08~2.28) 1.6E−02	2.2E−02 65.0%	1.12 (0.82~1.53) 4.7E−01
GAMT (19p13.3)	1.17 (0.81~1.70) 4.0E−01	0.69 (0.46~1.03) 6.6E−02	1.81 (1.07~ 3.06) 2.6E−02	0.54 (0.32~0.92) 2.2E−02	1.75 (1.22~2.51) 2.2E−03	< 1.0E−03 81.8%	1.08 (0.69~1.68) 7.5E−01

The contents of each cell represent estimated hazard ratio (HR), 95%CI of HR, and hypothesis testing *P* value of HR, and in each dataset, the samples are categorized into two groups using a best cutoff of expression level. Combined results are derived by meta-analysis with random effect model

^a^Expression data of the gene is not available in the corresponding GEO database

## Discussion

High-throughput sequencing technology, which has become cheaper, promotes the development of precision medicine [56]. Picking up underlying markers affecting disease prognosis from thousands of candidates calls for high-dimensional survival model besides generally used one-by-one Cox proportional hazards model. In this paper, we propose a parametric survival counterpart of EMVS, namely SurvEMVS, which employs a fast EM algorithm to fit all candidate biomarkers simultaneously and to explore posterior distribution of the unknown parameters, consequently to identify important signals and make effect estimations.

Much work has concentrated on developing new Bayesian methods on high-dimensional parametric survival model in application to medical or genetic data. For example, Sha et al. built AFT models with less common distribution (i.e., log-normal and log-t) for microarray data using a discrete spike-and-slab prior, where a time-consuming MCMC procedure was employed to simulate posterior distribution [57]. Mittal et al. developed four parametric models, i.e., exponential, Weibull, log-logistic, and log-normal distribution, by assigning Gaussian and Laplace prior to effects, where maximum a posterior (MAP) was used to acquire posterior modes of effects; however, this work lacked numerical study to evaluate their models, as well as discussion on variables selected in real data analysis from medical reasonability [58]. Newcombe et al. imposed a discrete spike-and-slab prior on coefficients of Weibull regression, where reversible jump MCMC being used for the posterior computation is ineffective. Moreover, it is unrepresentative of their application to a low-dimensional breast cancer data [59].

SurvEMVS imposes a continuous spike-and-slab mixture prior on effects to facilitate the separation of different effect sizes. This two-component prior can provide an indicator vector to guide variable selection whereby using a local version of the median probability model [22, 60]. In contrast, the EM algorithm or variational approximation employing one-component prior such as Laplace or *t* distribution does not involve variable inclusion indicators, and consequently makes variable selection indirectly [20]. Due to the unavailable closed form of maximization about maker effects in M-step, a variant of CCD algorithm serves to be feasible for obtaining approximate solutions. Consequently, our EM steps incorporating this fast CCD make the fitting much effective. One focus of this study is how to choose an optimal model from the hyperparameter tuning process. The EBIC, an extension of the BIC, is adopted with reason as follows: in comparison with the EBIC, the normally used AIC, BIC, or GCV would generate more spurious signal when applied to high-dimensional data, while CV-based metrics demand more computation and are unstable since the folds in CV are selected randomly. This is, to our knowledge, the first application of EBIC to the high-dimensional parametric survival analysis.

Over a range of simulation scenarios, our method with EBIC2 generally performs better than Cox LASSO in variable selection, parameter estimation, and prediction. In contrast, owing to imposing a single penalty on all effects, Cox LASSO yields high biased estimators. Our simulations also show the EBIC is appropriate for model selection, while the BIC (i.e., EBIC1) perform worst in situation with very large number of markers. For *p* > *n* problem in omics data, we recommend *τ* = 0.5 for EBIC in multi-stage study because it offers a good trade-off between the well-controlled FDR and the TPR and provides more opportunity to new findings. However, if one is strict to control false discovery, *τ* = 1, is recommended. Subsequently, we conducted two real data applications. In the first study with a lung cancer genotype data, 14 SNPs were detected by SurvEMVS, and further validation analyses using external data or function annotation resulted in 7 outstanding SNPs. In order to widen the application range of SurvEMVS considerably, we utilized a stomach cancer expression data in the second study. Expression levels of three genes are associated with cancer prognosis, and two of them are validated by extra GEO datasets along with one (namely SERPINE1) involved in tumor progression. The identification of well-known CTLA4 illustrates the availability of SurvEMVS. However, further functional experiments are needed for evidence of biologic plausibility of those identified markers.

Although we did not directly compare our EM algorithm with its MCMC counterpart, the speed advantage is apparent, according to the results of our EM algorithm under each optimal model converge needing 174 and 238 iterations in the two real data applications, which is much less than the chain length being set up for MCMC (usually > 10,000 for stable estimation). The model thus resembles a distinct iteration increase of real data application relative to a simulation study. We can explain that ideal conditions (e.g., sparse structure with independent markers) along with strong shrinkage of candidate hyperparameters (producing a parsimonious model) favor rapid convergence, but generally are not available under real data applications, which leads to more iterations demanding (but still fast enough) not only for SurvEMVS but also for other model [20].

However, we acknowledge that there are several limitations of the present study. First, SurvEMVS incorporating the EM, which seeks for a posterior mode rather than a whole posterior distribution of the parameter, cannot provide uncertain measure for estimators. We can directly disentangle this disadvantage by the bootstrap method, but have to bear expensive computations. Actually, another compromise may be adopted: the estimates of SurvEMVS can be considered as initial values of a following MCMC algorithm, which makes the MCMC procedure avoid the burn-in stage and finally yield fast and accurate estimators with uncertain measurement. Second, the most worrying thing of the parametric model is a situation of going against the parametric assumption for survival distribution. We show that SurvEMVS is robust for the status with moderately deviating from the Weibull premise. However, we believe that SurvEMVS will be less effective if the real survival time distinctly violates the Weibull distribution. We can bypass this limitation using a non-parametric AFT model like in [61], in which a Dirichlet process is used to make the model robust over a wider range of unknown baseline hazard. In addition, a lot of new directions for methodological work will arise from the current study. One obvious extension to our method will consider multivariate “*g*-priors” to reflect the effect correlations within the correlated markers [62]. Another interesting extension will involve introducing a newly developed spike-and-slab Laplace prior [63]. Going forward, the meaningful extension of SurvEMVS will integrate functional annotations or multi-omics data to powerfully mine association signals in future work.

## Conclusions

We present a new implementation of the EM algorithm for Bayesian variable selection under a Weibull survival model. Both of our simulation studies and two real data analyses show that the proposed method is effective and can cope with high-dimensional omics data.

## Additional file

Additional file 1: **Table S1.** TPR, FPR and FDR in variable selection with 50 replications (Weibull distribution). **Table S2.** TPR, FPR and FDR in variable selection with 50 replications (Gamma distribution). **Table S3.** Computational time (minutes) for application in simulation trials. **Table S4.** The estimated effects of 14 SNPs by SurvEMVS and classical Weibull regression. **Table S5.** Demographic and clinical characteristics of STAD patients. **Table S6.** The estimated effects of gene expression levels selected by SurvEMVS and Cox LASSO with their counterparts in low dimension scenario (i.e., Weibull regession and Cox model, respectively). **Figure S1.** Pseudocode for implementation of SurvEMVS. **Figure S2.** Averaged estimated effect (*black vertical lines*) for each marker over 50 replications under Scenario 2. **Figure S3.** Averaged estimated effect (*black vertical lines*) for each marker over 50 replications under Scenario 3. **Figure S4.** Averaged estimated effect (*black vertical lines*) for each marker over 50 replications under Scenario 4. **Figure S5.** Averaged estimated effect (*black vertical lines*) for each marker over 50 replications under Scenario 5. **Figure S6** Averaged estimated effect (*black vertical lines*) for each marker over 50 replications under Scenario 6. **Figure S7.** MSE of parameter estimation and AUC of prognosis prediction for Scenarios 3 and 4. **Figure S8.** MSE of parameter estimation and AUC of prognosis prediction for Scenarios 5 and 6. **Figure S9.** Kaplan-Meier survival curve of patients with high, moderate, and low risk. (DOCX 9932 kb)

## Abbreviations

AFT: Accelerated failure time
CCD: Cyclic coordinate descent
EBIC: Extended Bayesian Information Criteria
EM: Expectation-maximization
GEO: Gene Expression Omnibus
LASSO: Least absolute shrinkage and selection operator
MCMC: Markov Chain Monte Carlo
TCGA: The Cancer Genome Atlas

## References

[1] Metzker ML (2010). Sequencing technologies - the next generation. Nat Rev Genet.

[2] Veeramah KR, Hammer MF (2014). The impact of whole-genome sequencing on the reconstruction of human population history. Nat Rev Genet.

[3] Network TCGA (2014). Comprehensive molecular characterization of gastric adenocarcinoma. Nature.

[4] Edgar R, Domrachev M, Lash AE (2002). Gene expression omnibus: NCBI gene expression and hybridization array data repository. Nucleic Acids Res.

[5] Yang J, Lee SH, Goddard ME, Visscher PM (2011). GCTA: a tool for genome-wide complex trait analysis. Am J Hum Genet.

[6] Hu Z, Wu C, Shi Y, Guo H, Zhao X, Yin Z, Yang L, Dai J, Hu L, Tan W (2011). A genome-wide association study identifies two new lung cancer susceptibility loci at 13q12.12 and 22q12.2 in Han Chinese. Nat Genet.

[7] Dong J, Hu Z, Wu C, Guo H, Zhou B, Lv J, Lu D, Chen K, Shi Y, Chu M (2012). Association analyses identify multiple new lung cancer susceptibility loci and their interactions with smoking in the Chinese population. Nat Genet.

[8] Zhou X, Stephens M (2012). Genome-wide efficient mixed model analysis for association studies. Nat Genet.

[9] Chen H, Wang C, Conomos MP, Stilp AM, Li Z, Sofer T, Szpiro AA, Chen W, Brehm JM, Celedón JC (2016). Control for population structure and relatedness for binary traits in genetic association studies via logistic mixed models. Am J Hum Genet.

[10] Yang J, Zaitlen NA, Goddard ME, Visscher PM, Price AL (2014). Advantages and pitfalls in the application of mixed-model association methods. Nat Genet.

[11] Guan Y, Stephens M (2011). Bayesian variable selection regression for genome-wide association studies and other large-scale problems. Ann Appl Stat.

[12] Moser G, Sang HL, Hayes BJ, Goddard ME, Wray NR, Visscher PM (2015). Simultaneous discovery, estimation and prediction analysis of complex traits using a Bayesian mixture model. PLoS Genet.

[13] Tibshirani R (2011). Regression shrinkage and selection via the lasso: a retrospective. J R Stat Soc B.

[14] Zou H (2006). The adaptive lasso and its oracle properties. J Am Stat Assoc.

[15] Casella TP, George (2008). The Bayesian lasso. J Am Stat Assoc.

[16] George EI, Mcculloch RE (1997). Approaches for Bayesian variable selection. Stat Sin.

[17] Zhou X, Carbonetto P, Stephens M (2013). Polygenic modeling with Bayesian sparse linear mixed models. PLoS Genet.

[18] Carbonetto P, Stephens M (2012). Scalable variational inference for Bayesian variable selection in regression, and its accuracy in genetic association studies. Bayesian Anal.

[19] Logsdon BA, Carty CL, Reiner AP, Dai JY, Kooperberg C (2012). A novel variational Bayes multiple locus Z-statistic for genome-wide association studies with Bayesian model averaging. Bioinformatics.

[20] Duan W, Zhao Y, Wei Y, Yang S, Bai J, Shen S, Du M, Huang L, Hu Z, Chen F (2017). A fast algorithm for Bayesian multi-locus model in genome-wide association studies. Mol Gen Genet.

[21] Hayashi T, Iwata H (2010). EM algorithm for Bayesian estimation of genomic breeding values. BMC Genet.

[22] Ročková V, George EI (2014). EMVS: the EM approach to Bayesian variable selection. J Am Stat Assoc.

[23] Oakes D (2001). Biometrika centenary: survival analysis. Biometrika.

[24] Ziegel ER. Modelling for survival data in medical research by D. Collett: Chapman & Hall; 1994. https://www.crcpress.com/Modelling-Survival-Data-in-Medical-Research-Third-Edition/Collett/p/book/9781439856789.

[25] Hosmer DW, Lemeshow S. Applied survival analysis: regression modeling of time to event data: Wiley-Interscience; 1999. https://www.wiley.com/en-us/Applied+Survival+Analysis%3A+Regression+Modeling+of+Time+to+Event+Data%2C+2nd+Edition-p-9780471754992.

[26] Cox DR. Regression models and life-tables: Springer New York; 1992. https://link.springer.com/chapter/10.1007%2F978-1-4612-4380-9_37.

[27] Keiding N, Andersen PK, Klein JP (1997). The role of frailty models and accelerated failure time models in describing heterogeneity due to omitted covariates. Stat Med.

[28] Robins JM, Scheines R, Spirtes P, Wasserman L (2003). A Bayesian justification of Cox’s partial likelihood. Biometrika.

[29] Zucknick M, Saadati M, Benner A (2015). Nonidentical twins: comparison of frequentist and Bayesian lasso for Cox models. Biom J.

[30] Klein JP, Moeschberger ML (2003). Survival analysis: techniques for censored and truncated data.

[31] Dempster AP, Laird NM, Rubin DB (1977). Maximum likelihood from incomplete data via the EM algorithm. J R Stat Soc.

[32] Luenberger DG, Ye Y. Linear and nonlinear programming: Addison-Wesley; 1984. https://link.springer.com/book/10.1007%2F978-0-387-74503-9.

[33] Tong Z, Oles FJ (2001). Text categorization based on regularized linear classification methods. Inf Retr.

[34] Genkin A, Lewis DD, Madigan D (2007). Large-scale Bayesian logistic regression for text categorization. Technometrics.

[35] Simon N, Friedman J, Hastie T, Tibshirani R (2011). Regularization paths for Cox’s proportional hazards model via coordinate descent. J Stat Softw.

[36] Van Houwelingen HC, Bruinsma T, Hart AAM, Van'T Veer LJ, Wessels LFA (2006). Cross-validated cox regression on microarray gene expression data. Stat Med.

[37] Bogdan M, Ghosh JK, Doerge RW (2004). Modifying the Schwarz Bayesian information criterion to locate multiple interacting quantitative trait loci. Genetics.

[38] Siegmund D (2004). Model selection in irregular Problems: applications to mapping quantitative trait loci. Biometrika.

[39] Chen J, Chen Z (2008). Extended Bayesian information criteria for model selection with large model spaces. Biometrika.

[40] Jr HF, Lee KL, Mark DB (1996). Multivariable prognostic models: issues in developing models, evaluating assumptions and adequacy, and measuring and reducing errors. Stat Med.

[41] Purcell S, Neale B, Todd-Brown K, Thomas L, Ferreira MAR, Bender D, Maller J, Sklar P, Bakker PIWD, Daly MJ (2007). PLINK: a tool set for whole-genome association and population-based linkage analyses. Am J Hum Genet.

[42] Asomaning K, Miller DP, Liu G, Wain JC, Lynch TJ, Su L, Christiani DC (2008). Second hand smoke, age of exposure and lung cancer risk. Lung Cancer.

[43] Machida EO, Brock MV, Hooker CM, Nakayama J, Ishida A, Amano J, Picchi MA, Belinsky SA, Herman JG, Taniguchi S (2006). Hypermethylation of ASC/TMS1 is a sputum marker for late-stage lung cancer. Cancer Res.

[44] Zhao Y, Wei Q, Hu L, Chen F, Hu Z, Heist RS, Su L, Amos CI, Shen H, Christiani DC (2014). Polymorphisms in MicroRNAs are associated with survival in non-small cell lung cancer. Cancer Epidemiol Biomarkers Prev.

[45] Chandrashekar DS, Bashel B, Sah B, Creighton CJ, Ponce-Rodriguez I, Bvsk C, Varambally S (2017). UALCAN: a portal for facilitating tumor subgroup gene expression and survival analyses. Neoplasia.

[46] Brabender J, Danenberg KD, Metzger R, Schneider PM, Lord RV, Groshen S, Tsao-Wei DD, Park J, Salonga D, Holscher AH (2002). The role of retinoid X receptor messenger RNA expression in curatively resected non-small cell lung cancer. Clin Cancer Res.

[47] He S, Chen CH, Chernichenko N, He S, Bakst RL, Barajas F, Deborde S, Allen PJ, Vakiani E, Yu Z (2014). GFRα1 released by nerves enhances cancer cell perineural invasion through GDNF-RET signaling. Proc Natl Acad Sci U S A.

[48] Colaprico A, Silva TC, Olsen C, Garofano L, Cava C, Garolini D, Sabedot TS, Malta TM, Pagnotta SM, Castiglioni I (2016). TCGAbiolinks: an R/Bioconductor package for integrative analysis of TCGA data. Nucleic Acids Res.

[49] White IR, Royston P, Wood AM (2011). Multiple imputation using chained equations: issues and guidance for practice. Stat Med.

[50] Pardoll DM (2012). The blockade of immune checkpoints in cancer immunotherapy. Nat Rev Cancer.

[51] Wei SC, Levine JH, Cogdill AP, Zhao Y, Anang NAS, Andrews MC, Sharma P, Wang J, Wargo JA, Pe'er D (2017). Distinct cellular mechanisms underlie anti-CTLA-4 and anti-PD-1 checkpoint blockade. Cell.

[52] Hou R, Cao B, Chen Z, Li Y, Ning T, Li C, Xu C, Chen Z (2010). Association of cytotoxic T lymphocyte-associated antigen-4 gene haplotype with the susceptibility to gastric cancer. Mol Biol Rep.

[53] Kim JW, Nam KH, Ahn SH, Park DJ, Kim HH, Kim SH, Chang H, Lee JO, Kim YJ, Lee HS (2016). Prognostic implications of immunosuppressive protein expression in tumors as well as immune cell infiltration within the tumor microenvironment in gastric cancer. Gastric Cancer.

[54] Rakic JM, Maillard C, Jost M, Bajou K, Masson V, Devy L, Lambert V, Foidart JM, Noel A (2003). Role of plasminogen activator-plasmin system in tumor angiogenesis. Cell Mol Life Sci.

[55] Takayama Y, Hattori N, Hamada H, Masuda T, Omori K, Akita S, Iwamoto H, Fujitaka K, Kohno N (2016). Inhibition of PAI-1 limits tumor angiogenesis regardless of angiogenic stimuli in malignant pleural mesothelioma. Cancer Res.

[56] Collins FS, Varmus H (2015). A new initiative on precision medicine. N Engl J Med.

[57] Sha N, Tadesse MG, Vannucci M (2006). Bayesian variable selection for the analysis of microarray data with censored outcomes. Bioinformatics.

[58] Mittal S, Madigan D, Cheng JQ, Burd RS (2013). Large-scale parametric survival analysis. Stat Med.

[59] Newcombe P, Raza AH, Blows F, Provenzano E, Pharoah P, Caldas C, Richardson S (2014). Weibull regression with Bayesian variable selection to identify prognostic tumour markers of breast cancer survival. Stat Methods Med Res.

[60] Barbieri M, Berger J (2004). Optimal predictive model selection. Ann Stat.

[61] Zhang Zhen, Sinha Samiran, Maiti Tapabrata, Shipp Eva (2016). Bayesian variable selection in the accelerated failure time model with an application to the surveillance, epidemiology, and end results breast cancer data. Statistical Methods in Medical Research.

[62] Zellner A (1986). On assessing prior distributions and Bayesian regression analysis with G-prior distributions. Bayesian Inference Decis Tech.

[63] Ročková Veronika, George Edward I. (2016). The Spike-and-Slab LASSO. Journal of the American Statistical Association.

[64] Gao AC, Lou W, Isaacs JT (2000). Enhanced GBX2 expression stimulates growth of human prostate cancer cells via transcriptional up-regulation of the interleukin 6 gene. Clin Cancer Res.

[65] Gao AC, Lou W, Isaacs JT (1998). Down-regulation of homeobox gene GBX2 expression inhibits human prostate cancer clonogenic ability and tumorigenicity. Cancer Res.

[66] Nimmrich I, Erdmann S, Melchers U, Chtarbova S, Finke U, Hentsch S, Hoffmann I, Oertel M, Hoffmann W, Müller O (2001). The novel ependymin related gene UCC1 is highly expressed in colorectal tumor cells. Cancer Lett.

[67] Liu Z, Zhang J, Gao Y, Pei L, Zhou J, Gu L, Zhang L, Zhu B, Hattori N, Ji J (2014). Large-scale characterization of DNA methylation changes in human gastric carcinomas with and without metastasis. Clin Cancer Res.

[68] Godinheymann N, Brabetz S, Murillo MM, Saponaro M, Santos CR, Lobley A, East P, Chakravarty P, Matthews N, Kelly G (2015). Tumour-suppression function of KLF12 through regulation of anoikis. Oncogene.

[69] Yu N, Migita T, Hosoda F, Okada N, Gotoh M, Arai Y, Fukushima M, Ohki M, Miyata S, Takeuchi K (2009). Krüppel-like factor 12 plays a significant role in poorly differentiated gastric cancer progression. Int J Cancer.

[70] Rozenblum E, Vahteristo P, Sandberg T, Bergthorsson JT, Syrjakoski K, Weaver D, Haraldsson K, Johannsdottir HK, Vehmanen P, Nigam S (2002). A genomic map of a 6-Mb region at 13q21-q22 implicated in cancer development: identification and characterization of candidate genes. Hum Genet.

