# Supplementary material for: Bayesian variable selection for parametric survival model with applications to cancer omics data
Source: Hum Genomics. 2018 Nov 6;12:49. doi: 10.1186/s40246-018-0179-x (PMC6218990; doi:10.1186/s40246-018-0179-x)
Supplement: Supplementary file 1 — Table S1. TPR, FPR and FDR in variable selection with 50 replications (Weibull distribution). Table S2. TPR, FPR and FDR in variable selection with 50 replications (Gamma distribution). Table S3. Computational time (minutes) for application in simulation trials. Table S4. The estimated effects of 14 SNPs by SurvEMVS and classical Weibull regression. Table S5. Demographic and clinical characteristics of STAD patients. Table S6. The estimated effects of gene expression levels selected by SurvEMVS and Cox LASSO with their counterparts in low dimension scenario (i.e., Weibull regession and Cox model, respectively). Figure S1. Pseudocode for implementation of SurvEMVS. Figure S2. Averaged estimated effect (black vertical lines) for each marker over 50 replications under Scenario 2. Figure S3. Averaged estimated effect (black vertical lines) for each marker over 50 replications under Scenario 3. Figure S4. Averaged estimated effect (black vertical lines) for each marker over 50 replications under Scenario 4. Figure S5. Averaged estimated effect (black vertical lines) for each marker over 50 replications under Scenario 5. Figure S6 Averaged estimated effect (black vertical lines) for each marker over 50 replications under Scenario 6. Figure S7. MSE of parameter estimation and AUC of prognosis prediction for Scenarios 3 and 4. Figure S8. MSE of parameter estimation and AUC of prognosis prediction for Scenarios 5 and 6. Figure S9. Kaplan-Meier survival curve of patients with high, moderate, and low risk. (DOCX 9932 kb) [file 40246_2018_179_MOESM1_ESM.docx]

**Additional file**

**Supplementary Tables and Figures**

**Table** **S1.** **TPR, FPR and FDR in variable selection with 50 replications (Weibull distribution)**

| Method | **Scenario 3** (*p*=1000) | | |  | **Scenario 4** (*p*=5000) | | |
| --- | --- | --- | --- | --- | --- | --- | --- |
|  | TPR | FPR | FDR |  | TPR | FPR | FDR |
| LASSO.se | 0.800 | 1.55E-03 | 0.243 |  | 0.477 | 3.08E-04 | 0.350 |
| LASSO.min | 0.953 | 2.11E-02 | 0.785 |  | 0.770 | 4.55E-03 | 0.831 |
| EBIC($\tau$=0) | 0.757 | 5.84E-04 | 0.113 |  | 0.740 | 4.47E-03 | 0.834 |
| EBIC($\tau$=0.5) | 0.743 | 3.62E-04 | 0.075 |  | 0.527 | 1.80E-04 | 0.222 |
| EBIC($\tau$=1.0) | 0.713 | 2.82E-04 | 0.061 |  | 0.420 | 3.60E-04 | 0.067 |

Abbreviations: TPR, true positive rate; FPR, false positive rate; FDR, false discovery rate

**Table** **S2.** **TPR, FPR and FDR in variable selection with 50 replications (Gamma distribution)**

| Method | **Scenario 5** (*p*=1000) | | |  | **Scenario 6** (*p*=5000) | | |
| --- | --- | --- | --- | --- | --- | --- | --- |
|  | TPR | FPR | FDR |  | TPR | FPR | FDR |
| LASSO.se | 0.670 | 1.25E-03 | 0.236 |  | 0.333 | 1.52E-04 | 0.275 |
| LASSO.min | 0.890 | 1.69E-02 | 0.759 |  | 0.690 | 4.58E-03 | 0.847 |
| EBIC($\tau$=0) | 0.760 | 9.26E-04 | 0.168 |  | 0.687 | 6.00E-03 | 0.879 |
| EBIC($\tau$=0.5) | 0.687 | 5.84E-04 | 0.123 |  | 0.513 | 2.12E-04 | 0.256 |
| EBIC($\tau$=1.0) | 0.687 | 4.63E-04 | 0.100 |  | 0.320 | 8.01E-04 | 0.020 |

Abbreviations: TPR, true positive rate; FPR, false positive rate; FDR, false discovery rate

**Table S3. Computational time (minutes) for application in simulation trials**

| Data Dimensionality | Scenario | SurvEMVS | Cox LASSO |
| --- | --- | --- | --- |
| *n*=500, *p*=1,000 | 1 (Exponential) | 0.890(0.040) | 0.191(0.006) |
|  | 3 (Weibull) | 0.734(0.046) | 0.182(0.005) |
|  | 5 (Gamma) | 0.841(0.030) | 0.227(0.006) |
| *n*=500, *p*=5,000 | 2 (Exponential) | 3.571(0.171) | 0.517(0.034) |
|  | 4 (Weibull) | 3.278(0.173) | 0.619(0.022) |
|  | 6 (Gamma) | 3.553(0.127) | 0.910(0.034) |

Time for simulation trials is averaged over 50 replications. The corresponding standard deviation is shown in parentheses.

**Table S4.** **The estimated effects of 14 SNPs by SurvEMVS and classical Weibull regression**

| SNPs | SurEMVS^a^ | Weibull^b^ | SNPs | SurEMVS^a^ | Weibull^b^ |
| --- | --- | --- | --- | --- | --- |
| rs1506943_G | -0.088 | -0.094 | rs2044831_G | -0.145 | -0.161 |
| rs16865111_C | 0.096 | 0.099 | rs9986932_A | -0.135 | -0.150 |
| rs1489701_G | -0.110 | -0.112 | rs263264_G | -0.093 | -0.095 |
| rs1921660_G | -0.132 | -0.147 | rs2074986_G | -0.085 | -0.088 |
| rs981852_C | -0.108 | -0.116 | rs4885110_A | 0.138 | 0.140 |
| rs12651293_T | -0.118 | -0.125 | rs1188568_G | -0.126 | -0.130 |
| rs7783753_T | -0.110 | -0.117 | rs169143_G | 0.113 | 0.112 |

^a^ The estimates of 14 SNPs are picked up from the full model with 3,911 SNPs and clinical variables fitted by SurvEMVS.

^b^ Only 14 SNPs and clinical variables are fitted by Weibull regression.

**Table** **S5.** **Demographic and clinical characteristics of STAD patients**

| Characteristic | STAD samples (N=415) | No. of Missing |
| --- | --- | --- |
| Censor rate | 61.2% |  |
| Age, mean years (SD) | 66.2 (10.7) | 9 |
| Gender (male vs. female) | 286/147 | 0 |
| Race (white vs. others) | 260/100 | 55 |
| Pstage (early[I-II] vs. late[III-IV]) | 180/210 | 25 |
| Grade (gx/g1/ g2/ g3) | 9/12/148/246 | 0 |

**Table S6**. **The estimated effects of gene expression levels selected by SurvEMVS and Cox LASSO with their counterparts in low dimension scenario (i.e. Weibull regession and Cox model, respectively)**

| Gene Symbol | SurvEMVS | Weibull | Cox LASSO | Cox |
| --- | --- | --- | --- | --- |
| CTLA4  (2q33.2) | 0.295 | 0.304 | -0.007 | -0.251 |
| PLCXD3  (5p13.1) | 一^a^ | 一 | 0.00027 | 0.165 |
| NACAD  (7p13) | -0.156 | -0.142 | 一 | 一 |
| SERPINE1  (7q22.1) | -0.277 | -0.268 | 0.027 | 0.258 |
| ALG11  (13q14.3) | 一 | 一 | -0.116 | -0.221 |
| GAMT  (19p13.3) | 一 | 一 | 0.015 | 0.212 |

^a^ The gene is not selected by the corresponding model.

| **Algorithm**: SurvEMVS   - Inputs: $X,Z,T,\delta,\upsilon_{0},\upsilon_{1},a,b,\nu,\eta,\xi,k',\beta'$; - Initialize ; - For *k*=1,2,… until convergence: - E-step: Update formula (3) and (4). - M-step:   (1) Update  with a variant of CCD algorithm:  For $j\in\left\{1,2,...,p\right\}$ if $k\leq k'$,  if $k>k'$,   - Compute first (*L*_1_) and second derivate (*L*_2_) of formula(5) at ; - ; - ; - ; - ;   Next *j*.  (2) Update  by formula (6)-(9). |
| --- |

**Figure S1.** **Pseudocode for implementation of SurvEMVS.**


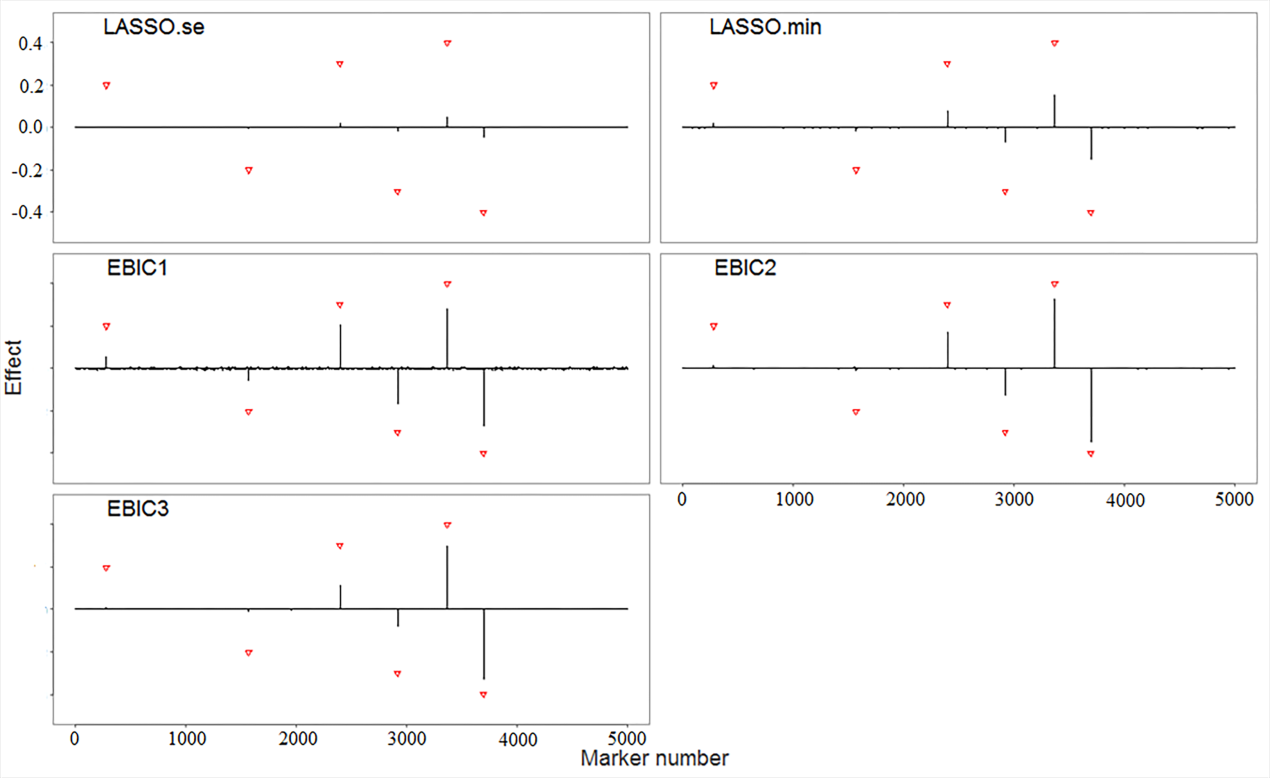


**Figure S2.** **Averaged estimated effect (*black vertical lines*) for each marker over 50 replications under** **Scenario 2**. Red triangles label true effect sizes and locations of the causal markers.

**
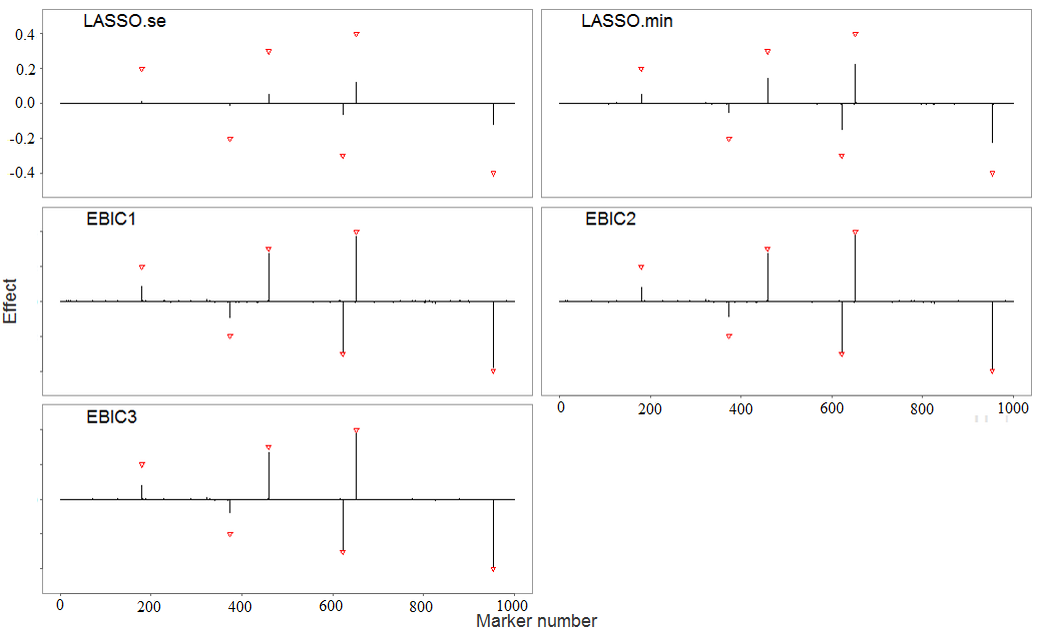
**

**Figure S3.** **Averaged estimated effect (*black vertical lines*) for each marker over 50 replications under** **Scenario 3**. Red triangles label true effect sizes and locations of the causal markers.

**
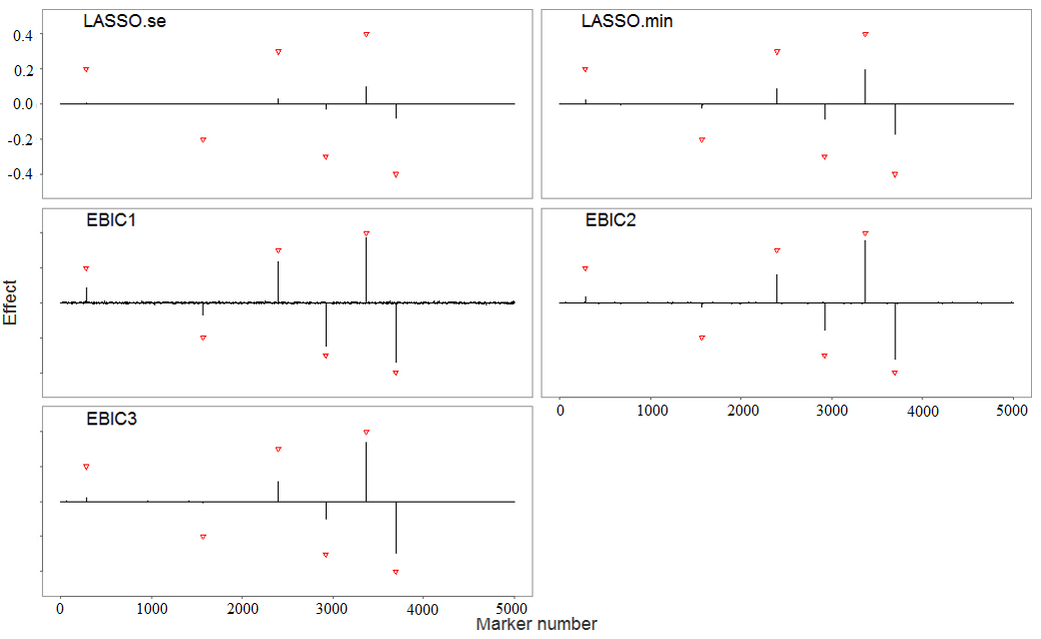
**

**Figure S4.** **Averaged estimated effect (*black vertical lines*) for each marker over 50 replications under** **Scenario 4**. Red triangles label true effect sizes and locations of the causal markers.

**
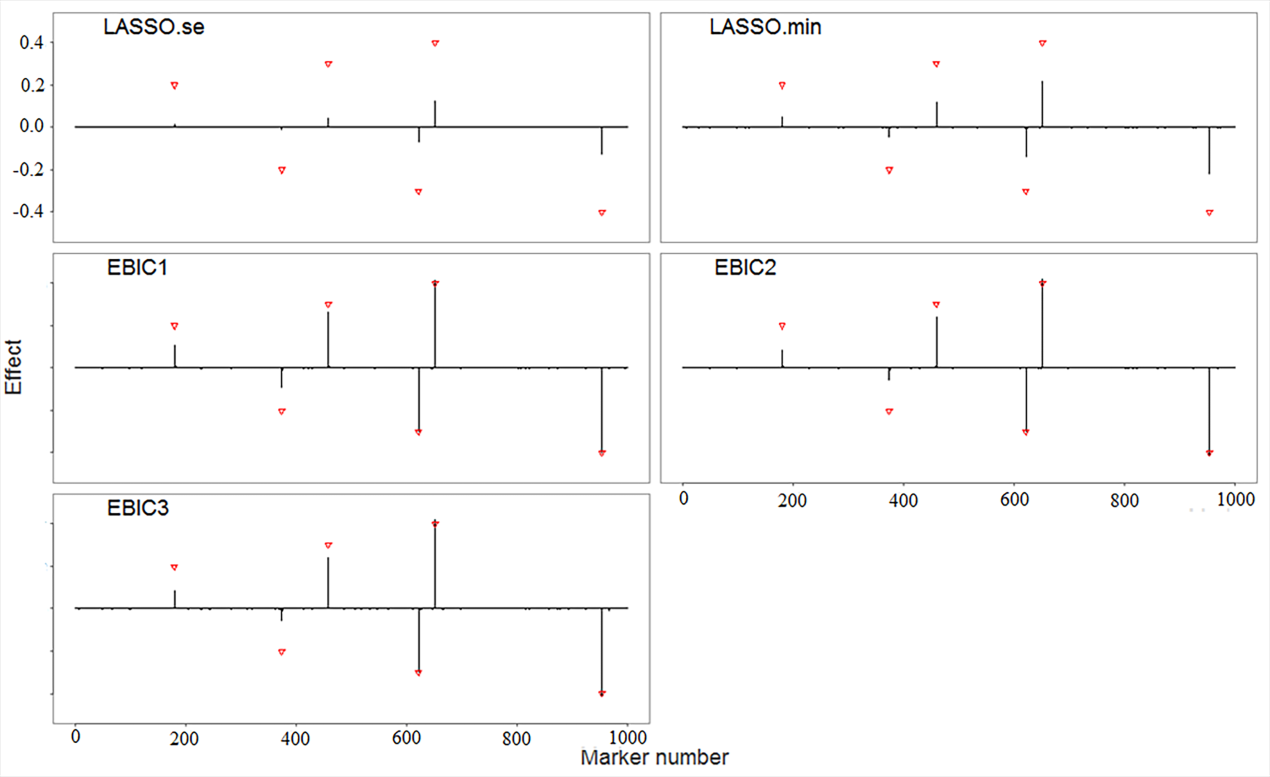
**

**Figure S5**. **Averaged estimated effect (*black vertical lines*) for each marker over 50 replications under Scenario 5.** Red triangles label true effect sizes and locations of the causal markers.

**
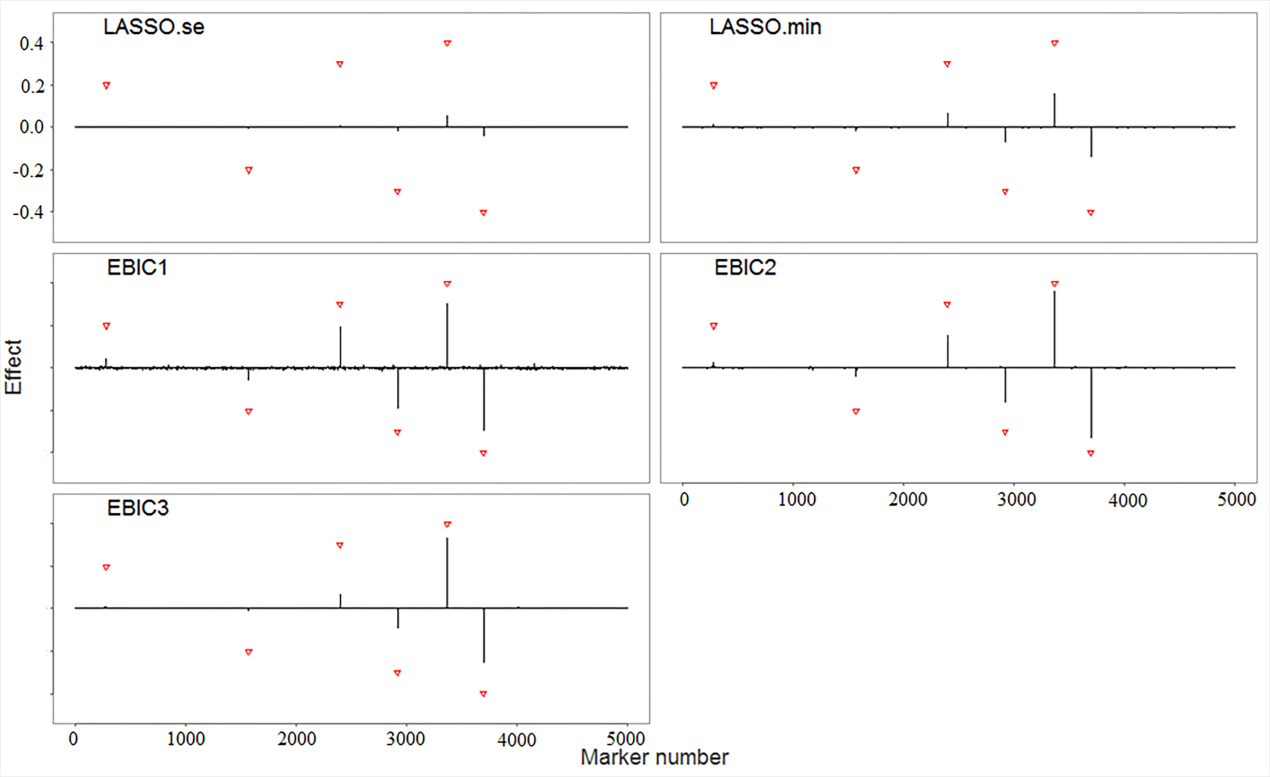
**

**Figure S6.** **Averaged estimated effect (*black vertical lines*) for each marker over 50 replications under Scenario 6.** Red triangles label true effect sizes and locations of the causal markers.

**
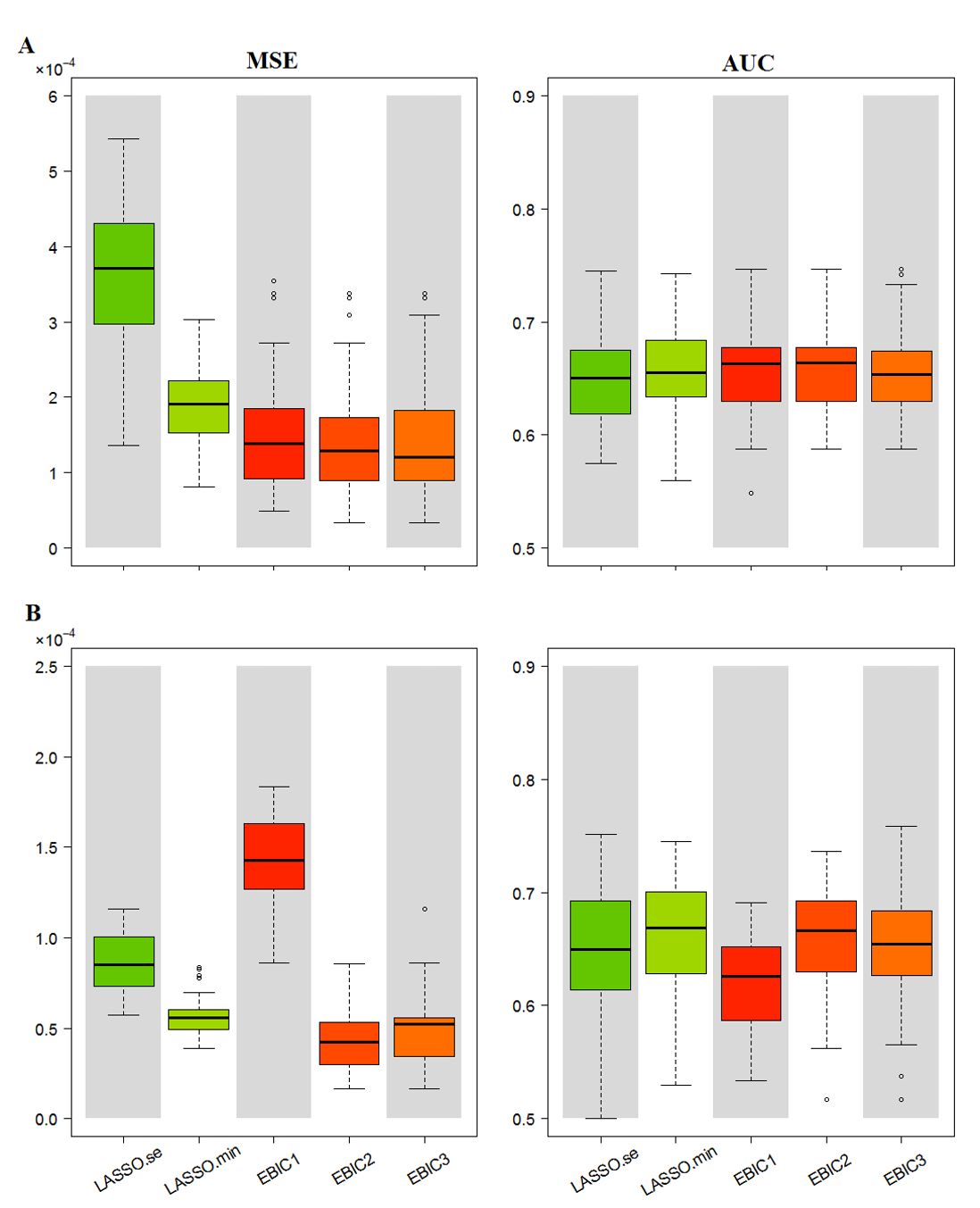
**

**Figure S7.** **MSE of parameter estimation and AUC of prognosis prediction for Scenario 3 and 4.** Panel A and B represent the results of Scenario 3 and 4, respectively.

**
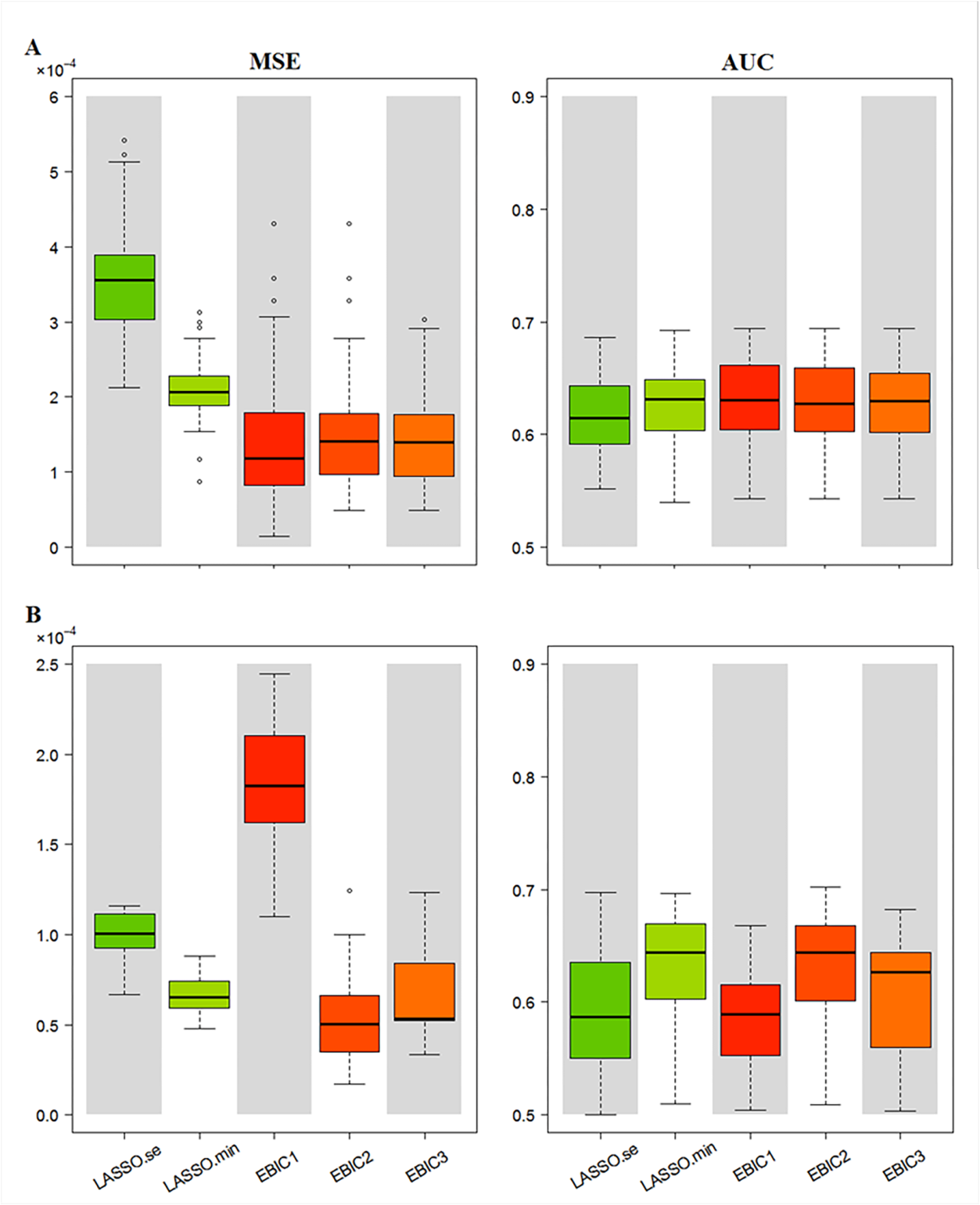
**

**Figure S8.** **MSE of parameter estimation and AUC of prognosis prediction for Scenario 5 and 6.** Panel A and B represent the results of Scenario 5 and 6, respectively.

**
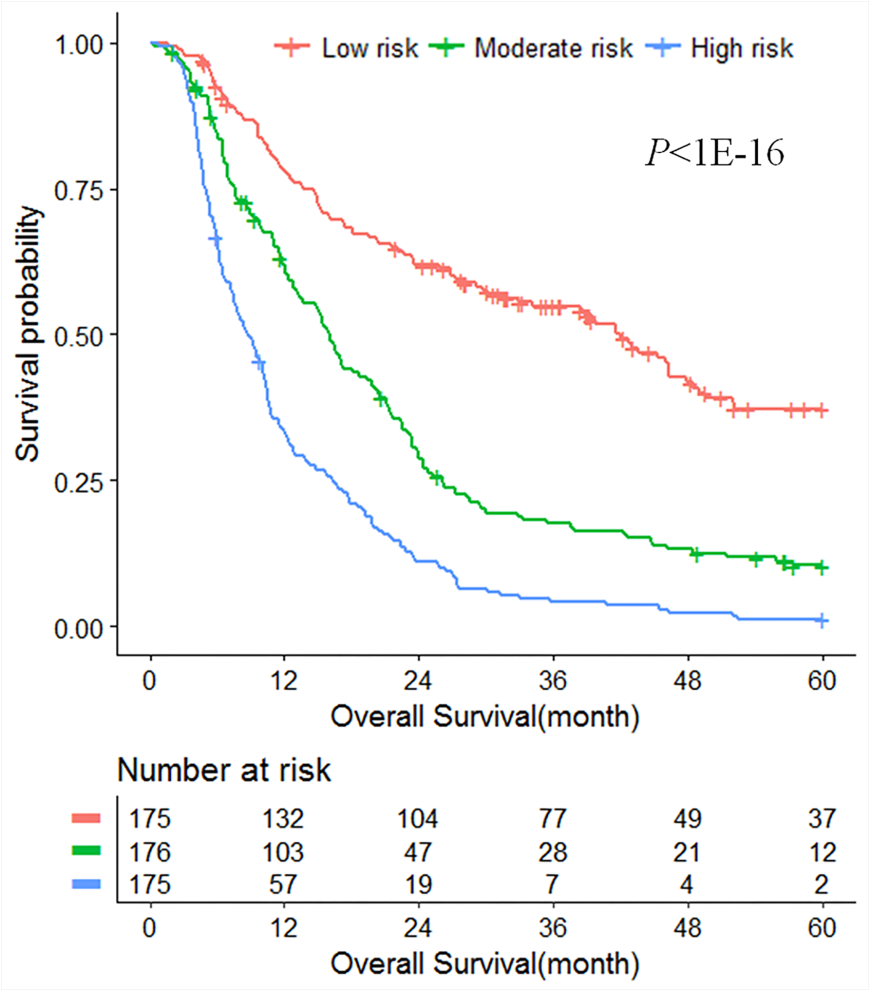
**

**Figure S9.** **Kaplan-Meier survival curve of patients with high, moderate, and low risk.** *P* value is calculated using log-rank test.
